# Occurrence and transformation of phenoxy acids in aquatic environment and photochemical methods of their removal: a review

**DOI:** 10.1007/s11356-019-06510-2

**Published:** 2019-12-01

**Authors:** Paweł Muszyński, Marzena S. Brodowska, Tadeusz Paszko

**Affiliations:** 1 Department of Chemistry, University of Life Sciences in Lublin, Akademicka Street 15, 20-950 Lublin, Poland; 2 Department of Agricultural and Environmental Chemistry, University of Life Sciences in Lublin, Akademicka Street 15, 20-950 Lublin, Poland

**Keywords:** Herbicides, Water biodegradation, Photodegradation, Photocatalytic degradation, Phenoxy acids, Water

## Abstract

The article presents the behavior of phenoxy acids in water, the levels in aquatic ecosystems, and their transformations in the water environment. Phenoxy acids are highly soluble in water and weakly absorbed in soil. These highly mobile compounds are readily transported to surface and groundwater. Monitoring studies conducted in Europe and in other parts of the world indicate that the predominant phenoxy acids in the aquatic environment are mecoprop, 4-chloro-2-methylphenoxyacetic acid (MCPA), dichlorprop, 2,4-dichlorophenoxyacetic acid (2,4-D), and their metabolites which are chlorophenol derivatives. In water, the concentrations of phenoxy acids are effectively lowered by hydrolysis, biodegradation, and photodegradation, and a key role is played by microbial decomposition. This process is determined by the qualitative and quantitative composition of microorganisms, oxygen levels in water, and the properties and concentrations of phenoxy acids. In shallow and highly insolated waters, phenoxy acids can be decomposed mainly by photodegradation whose efficiency is determined by the form of the degraded compound. Numerous studies are underway on the use of advanced oxidation processes (AOPs) to remove phenoxy acids. The efficiency of phenoxy acid degradation using AOPs varies depending on the choice of oxidizing system and the conditions optimizing the oxidation process. Most often, methods combining UV radiation with other reagents are used to oxidize phenoxy acids. It has been found that this solution is more effective compared with the oxidation process carried out using only UV.

## Introduction

**Fig. 1:**
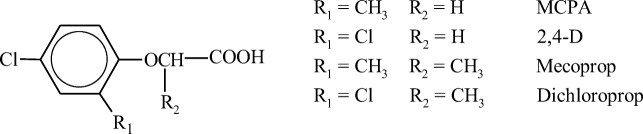
Structure of phenoxyalkane acids (based on Zertal et al. 2004)

Herbicides from the phenoxyalkane acid group are the oldest yet still widely applied weed control agents. The main active ingredients in herbicide formulations include derivatives of phenoxyacetic acid: 4-chloro-2-methylphenoxyacetic acid (MCPA) and 2,4-dichlorophenoxyacetic acid (2,4-D) and phenoxypropionic acid (mecoprop (MCPP) and dichlorprop (DCPP)), and, less frequently, phenoxybutanoic acid derivatives (2,4-dichlorophenoxybutanoic acid (2,4-DB) and 4-chloro-2-methylphenoxybutanoic acid (MCPB)). These compounds contain a substituted aromatic ring linked with a carboxylic acid residue via an ether bond (Fig. 1). Carbon–chlorine bonds and carbon–methyl group bonds in the aromatic ring are important structural elements that influence the reactivity and lipophilicity of phenoxy acids. The C–Cl bond is highly stable due to the coupling of chlorine atom electrons with π-electrons of the aromatic ring. Mecoprop and dichlorprop are chiral compounds consisting of two isomeric molecules (S and R enantiomers) that differ in the spatial arrangement of atoms. The R enantiomer is the only biologically active enantiomeric form of mecoprop and dichlorprop (Buser and Müller 1997; Müller and Buser 1997).

Mecoprop and dichlorprop are present in many formulations as enantiomerically pure compounds mecoprop-P and dichlorprop-P; however, racemic mixtures are still being used. MCPA, 2,4-D, (R)-mecoprop, and (R)-dichlorprop formulations are selective herbicides that are used in crop fields (in particular cereals), orchards, and meadows, as well as forests, gardens, and water bodies (as salts and esters) (Li et al. 2003; Kwan and Chu 2004; US EPA 2005; Matamoros et al. 2012; Yuzir et al. 2013; Song 2014; NIPC 2015; Peterson et al. 2016). The structure and activity of these compounds are similar to those of the natural hormone indole-3 acetic acid (IAA) (auxin) (Roberts et al. 1998; Venkov et al. 2000; Buss et al. 2006; Mithila et al. 2011). At higher concentrations, phenoxy acids induce rapid, uncontrolled growth of dicotyledonous plants, which leads to plant death (Holland et al. 2002; Chu et al. 2004; Cassanego et al. 2010; Song 2014; NIPC 2015; Islam et al. 2017).

Phenoxy acids are produced in three forms: salts (alkaline, amine), esters, and acids. All forms are soluble in water, but salts exhibit the highest solubility (follow Tayeb et al. 2011). Phenoxy acid salts and acidic forms dissociate in water and form anions of parent phenoxy acids. In turn, the hydrolysis of esters depends on time and pH value (Waite et al. 2002). Due to their high solubility in water and low sorption in soil, phenoxy acids are characterized by high mobility in soils, especially in sandy soils with low organic matter content. Therefore, they can penetrate to groundwater and surface water (US EPA 2005; Hiller et al. 2010; PPDB 2013; Sanchis et al. 2014; López-Piñeiro et al. 2019). However, the ability of phenoxy acids to penetrate the groundwater level is limited by their relatively high degradation rate (Boivin et al. 2005). Their content in water varies because it is dependent on various factors, including the contamination source and its distance from water and soil, climatic and geological factors, herbicide type, and frequency of application. Phenoxy acid concentrations in groundwater are substantially lower than that in surface water (ng–μg L^−1^) (Kolpin et al. 2000; Thorling et al. 2012). Ground and surface water are sources of drinking water. Phenoxy acids are endocrine active compounds that are absorbed from the gastrointestinal tract into the human body. Drinking water containing phenoxy acids is a significant health risk. This problem occurs in countries that have limited water resources, as well as in the case of the use of inefficient purification processes, such as open aeration and filtration (Jørgensen and Stockmarr 2009). The European Commission (EC) has set limits for pesticides in groundwater, which are 0.1 μg L^−1^ for the compound or its metabolite and 0.5 μg L^−1^ for the sum of parent compounds and their metabolites (Scheidleder et al. 1999; EC 2006), while EC has not yet set limits on the content of phenoxy acids in surface waters. Concentration limits in these waters concern hazardous priority substances whose presence in the waters of the countries of the Community should be constantly monitored and to which the phenoxy acids are not included. Directive 2008/105/EC of the European Parliament and of the Council (European Union 2008) has provided information that the EC is considering the possibility of including mecoprop in the list of priority substances. Until now, in no EC regulation on environmental water quality, there is no confirmation that mecoprop has been classified as a priority substance. As previously mentioned, surface waters are sources of drinking water. Therefore, in the literature of monitoring of surface waters, examples of comparison of concentrations of phenoxy acids in these waters with a limit set for drinking water can be found. According to the US EPA (2015), the maximum concentration of 2,4-D in drinking water should not exceed 70 μg L^−1^.

## Phenoxy acid levels in groundwater and surface water

The problem of water contamination with organic compounds is continuously monitored in most EU countries. In 2008, groundwater purity from 23 European countries was compared. Mecoprop (13.4%), MCPA (7.9%), dichlorprop (4.9%), and 2,4-D (3.7%) were the most ubiquitous phenoxy acids (Table 1). In some samples, the concentrations of mecoprop and dichlorprop exceeded 0.1 μg L^−1^ (EA 2003; EHS 2005; Loos et al. 2010). In Poland, a water monitoring survey carried out in agricultural areas near Wrocław (Sadowski and Kucharski 2006) revealed that MCPA, MCPP, and 2,4-D were present at concentrations ≥ 0.1 μg L^−1^ in approximately 7%, 1.5%, and 0.8% of the analyzed samples. In the work of Buczyńska and Szadkowska-Stańczyk (2005), MCPA, MCPP, and 2,4-D concentrations in groundwater also exceeded 0.1 μg L^−1^. Metabolites of phenoxy acid are also present in groundwater. In Denmark, 2,4-dichlorophenol was found in 10% of the analyzed samples (Juhler and Felding 2003). In Ireland, 4-chloro-2-methylphenol (4-C2MP) was the most common contaminant (26% of samples), whereas 2,4-dichlorophenol was less frequently noted (around 13% of samples) (Richards 2013). The concentrations of 4-C2MP were below 0.1 μg L^−1^ in most of the analyzed samples, whereas 2,4-dichlorophenol concentrations did not exceed the limit for drinking water.

**Table 1:** Summary of the groundwater and river water monitoring for phenoxy acids and in EU countries

Chemical	Limit of detection [μg L^−1^]	Frequency of detection [%]	Max concentration [μg L^−1^]	Average concentration [μg L^−1^]	90th percentile [%]	Reference
Groundwater (number of samples 164)						
Mecoprop	0.2	13.4	0.785	0.007	1	Loos et al. (2010)
MCPA	0.1	7.9	0.036	0	0	
Dichlorprop	0.1	4.9	3.199	0.036	0	
2,4-D	0.1	3.7	0.012	0	0	
River water (number of samples 122)						
Mecoprop	0.1	43	0.194	0.015	0.054	Loos et al. (2009)
2,4-D	0.1	52	1.221	0.022	0.035	

Loos et al. (2009) investigated the water pollution with polar compounds of rivers in 27 European countries, including Poland (Table 1). The presence of 2,4-D was found in 52% of the analyzed samples, and mecoprop was detected in 43% of the samples. The concentrations of both compounds exceeded the EU (European Union) limit, and mean concentrations were estimated at 0.022 μg L^−1^ and 0.015 μg L^−1^, respectively (Loos et al. 2009). In Poland (Krzyżanowski 2008; Sadowski et al. 2009; Sadowski et al. 2014), MCPA and 2,4-D were the most frequently detected phenoxy acids, but they exceeded the EU standards only sporadically.

Contamination of surface water and groundwater with phenoxy acids poses a problem not only for European countries (Ignatowicz-Owsieniuk and Skoczko 2002 (Poland); Köck et al. 2010 (Spain); Roseth 2013 (Norway); McManus et al. 2014 (Ireland); McKnight et al. 2015 (Denmark); Székács et al. 2015 (Hungary)) but it is a global scale problem. Phenoxy acids were found in water resources of Canada (Kurt-Karakus et al. 2010; Glozier et al. 2012), the USA (Ensminger et al. 2013; DaSilva 2016), and Australia (Schult 2016).

Seasonal differences in phenoxy acid levels in water are primarily associated with the frequency and timing of herbicide treatments in agriculture. They also depend on herbicide doses and climatic factors (Comoretto et al. 2008). Raina et al. (2011) reported higher concentrations of mecoprop, 2,4-D, and MCPA in spring and summer months. In Poland, herbicides containing MCPA and 2,4-D are applied in spring and autumn, and these compounds are most frequently detected in water samples collected during these seasons (Ignatowicz and Struk-Sokołowska 2004; Sadowski and Kucharski 2006; Sadowski et al. 2009). Phenoxy acid concentrations may also fluctuate naturally across the seasons of the year. In the temperate zone, these compounds may accumulate in surface waters (such as lakes) in winter due to lower biodegradation rate (low water temperature) and lower efficiency of photolysis (short day).

## Biodegradation

Microbial degradation plays a significant role in phenoxy acid transformations in water. Phenoxy acids are degraded by aerobic and anaerobic bacteria. The efficiency of biodegradation is determined by the properties of the decomposed compound and factors that influence microbial growth, including water temperature, pH, oxygen, phosphorus, and nitrogen concentrations (Nesbitt and Watson 1980; Ghassemi et al. 1981). In comparison with surface water, groundwater is characterized by constant temperature, relatively low content of organic matter and bacteria (10^2^–10^4^ cells mL^−1^ groundwater and 10^3^–10^6^ cells g^−1^ sediment) (Albrechtsen and Winding 1992; Dobbins et al. 1992; fallow Tuxen 2002), and higher content of dissolved mineral compounds (Satora and Kaczor 2006).

The adsorption by bottom sediments influences the availability of herbicides for microorganisms and also has an impact on the level of their content in water. The content of organic matter and the type, quantity, and pH of mineral components in sediments play key roles in phenoxy acid adsorption onto sediments. The accumulation of phenoxy acids in sediments is also determined by their form, concentration, and solubility, as well as water pH. The phenoxy acids are characterized by low or medium hydrophobicity and, therefore, have low capacity for accumulation in bottom sediments (Caux et al. 1995; Gamhewage et al. 2019). According to Albrechtsen et al. (1996), the number of microorganisms in sediments is one of the factors that determines whether effective degradation of organic compounds will be possible. In the studies of Levi et al. (2014), it has been proved that degradation of mecoprop and dichlorprop at environmentally relevant concentrations (1 μg L^−1^) may occur in bottom sediments. The degradation process of these compounds stimulated the addition of oxygen. It was found that ^14^C-mecoprop was mineralized up to 27% with oxygen concentration of 1.4 mg O_2_ g^−1^ dw (dry weight) sediment. It was shown that dichloroprop was less degraded than mecoprop. Bottom sediments can consume oxygen, e.g., for oxidation of Mn(II) or Fe(II) (Tuxen et al. 2006; Levi et al. 2014), and in that way limit the availability of oxygen needed for biodegradation.

Biodegradation is often preceded by the adaptation of microorganisms to the presence of phenoxy acids in the environment (lag phase period). The degradation rate in the adaptive period is minimal. In a study by Meylan and Howard (1991), a lag phase for dichlorprop lasted several months, whereas Klingt et al. (1993) observed the lag phase of 35–40 days for mecoprop in groundwater. Mixtures of mecoprop and dichlorprop enantiomers may undergo biodegradation with two lag phases during which bacteria adapt to different optical isomers (Zipper et al. 1999). The lag period is shorter when the biodegradation process involves bacteria that had previous contact with phenoxy acids (de Lipthay et al. 2000; Toräng et al. 2000).

According to the literature, chlorophenols are the primary products of phenoxy acid degradation (2,4-dichlorophenol for 2,4-D and dichlorprop) under aerobic conditions, whereas 4-chloro-2-methylphenol (4-C2MP, also known as chlorocresol and p-chloro-o-cresol (PCOC)) is the primary product of MCPA and mecoprop degradation (Reitzel et al. 2004; US EPA 2007). Aerobic biodegradation of mecoprop and dichlorprop is dependent on their composition, i.e., whether these compounds occur as pure enantiomers or racemic mixtures. The rate at which enantiomerically pure compounds or their racemic mixtures are decomposed can differ for each enantiomer under various conditions (Casas et al. 2017). In a study analyzing aerobic biodegradation of racemic dichlorprop in sewage, the (S)-isomer was decomposed at a faster rate than the (R)-enantiomer (Zipper et al. 1999). In contrast, the (R)-isomer was degraded at a faster rate than the (S)-isomer in marine water (Ludwig et al. 1992). Buser and Müller (1998) demonstrated that (R)-mecoprop can be converted to (S)-mecoprop and (R)-dichlorprop can be converted to (S)-dichlorpop. Changes in the quantitative ratio of the (R)-isomer to the (S)-isomer of mecoprop were also reported by Heron and Christensen (1992) and Zipper et al. (1998). The biodegradation process of MCPB is different. The first biodegradation step involves β-oxidation during which the MCPB hydrocarbon chain is shortened by 2 carbon atoms to produce 2-methyl-4-chlorophenoxyacetic acid (Smith and Hayden 1981).

Microorganisms have to slowly adapt to the presence of phenoxy acids in aquatic environments. One of the adaptive mechanisms involves the expression of the gene encoding the synthesis of enzymes that decompose the pollutant. For example, the presence of 2,4-D induces the transcription of tfdA genes in aquatic microorganisms (Batıoglu-Pazarbaş et al. 2012; Batıoglu-Pazarbaş et al. 2013). These genes encode dioxygenases, the main enzymes that participate in aerobic degradation of aromatic compounds. In the presence of dioxygenases, 2,4-D is transformed to 2,4-dichlorophenol (de Lipthay et al. 2002). The quantitative and qualitative composition of aquatic microflora is modified by its exposure to mixtures of herbicides.

Mixtures of herbicides can affect both the number of water microflora and also modify its qualitative composition. De Lipthay et al. (2003) analyzed the influence of an herbicide mixture (containing mecoprop and dichlorprop) on microbial diversity in sediments and groundwater in the water-bearing horizon under aerobic conditions. Sediment and water samples were collected from segments of the water-bearing horizon exposed to low herbicide concentrations (< 40 μg L^−1^) as well as non-exposed segments. The presence of a heterogeneous population of phenoxy acid–degrading microorganisms in samples exposed to a mixture of herbicides has been found. In addition, in the species composition of these microorganisms, the share of bacteria from the genus *Pseudomonas* has increased.

According to the literature, phenoxy acids are stable under anaerobic conditions (Harrison et al. 1998; Albrechsten et al. 2001; Albrechsten et al. 2003), and if they are decomposed, the degradation process is very slow (Walters 1999; Howard 1991). The 2,4-dichlorophenol (DCP) metabolite is also decomposed more slowly under anaerobic conditions than in the presence of oxygen. Bacteria have fewer sources of energy under anaerobic conditions, which decreases the efficiency and rate of anaerobic degradation. Many studies investigating the efficiency of biodegradation in environment with limited oxygen access were conducted in the water-bearing horizon (Rügge et al. 1999; Arildskov et al. 2001). The top layer of the water-bearing horizon may contain limited amounts of oxygen from infiltrating rain water, but deeper strata are progressively deficient in oxygen (Pedersen et al. 1991; Pedersen 2000). For this reason, degradation processes in the water-bearing horizon are determined by redox conditions and the availability of electron acceptors other than oxygen for microorganisms, including nitrates(V), sulfates(VI), and Fe^3+^. In a study by Zipper et al. (1999), 2,4-D was decomposed under anaerobic conditions, but no decomposition was reported for mecoprop and dichlorprop. In an oxygen-deficient environment, 2,4-D was degraded due to breakage of the hydrocarbon chain or dechlorination. The by-product of dechlorination is 4-chlorophenoxyacetic acid (4-CPA) which is synthesized when a chlorine atom is substituted with a hydrogen atom (Boyle et al. 1999; Stotzky and Bollag 2000). Anaerobic bacteria utilizing various electron acceptors differ in their potential to degrade phenoxy acids. Larsen and Aamand (2001) observed low levels of mecoprop degradation in water samples from the water-bearing horizon under denitrification or methanogenic conditions. Mecoprop was not decomposed in groundwater from aquifer under sulfur-reducing conditions. Williams et al. (2003) investigated whether microbial degradation of mecoprop was an enantioselective process and whether redox conditions influence the stereochemistry of biodegradation. They analyzed groundwater samples from a landfill in Lincolnshire Limestone (UK) and observed that redox conditions were responsible for differences in the proportions of (R)- and (S)-isomers of mecoprop. In samples collected from sites situated 500–900 m from the landfill, which were characterized by nitrate(V) and Fe-reducing conditions, the (S)-isomer was predominant in the enantiomer mixture. Samples collected from aerobic water deposits situated 1000–2650 m from the landfill were characterized by higher concentration of the (R)-isomer and reduced levels of the (S)-isomer. In the most remote site, the concentrations of both enantiomers were identical. In water samples collected further than 3000 m from the landfill, where sulfate(VI) reducing conditions were present, the content of the (S)-isomer clearly exceeded the concentration of the (R)-isomer. The results of the field study were confirmed in a microcosm test in a laboratory. Both enantiomers were degraded under aerobic conditions, but the (S)-isomer was degraded significantly faster (zero-order reaction rate constant = 1.9 mg L^−1^ day^−1^) than the (R)-isomer (zero-order reaction rate constant = 1.32 mg L^−1^ day^−1^). However, in the presence of nitrates(V), degradation of the (R)-isomer occurred (zero-order reaction rate constant = 0.65 mg L^−1^ day^−1^). The energy gain of bacteria utilizing nitrates(V), sulfates, and Fe^3+^ differs. The gain is highest for denitrification bacteria which therefore have a higher potential for degrading phenoxy acids.

The concentration of the compound is one of the factors that affects the course of microbiological degradation (Janniche et al. 2010). Toräng et al. (2003) reported a significantly higher rate of mecoprop degradation in aerobic samples from the water-bearing horizon with high initial concentrations of mecoprop (25–100 μg L^−1^) than in samples where initial mecoprop levels were low (1–10 μg L^−1^). These studies (Toräng et al. 2003) show that there is a certain threshold concentration different for phenoxy acids (2,4-D and MCPP) below which the growth of the microbial population is latched. The effect of this is the constant and slow degradation rate of the phenoxy acid. Such degradation is also a characteristic at low concentrations of these compounds. The activity of bacteria in relation to individual phenoxy acids, even of similar structure, is not identical. Therefore, the threshold concentration value shows differentiation for different bacterial strains and also depends on physicochemical properties of water (Gözdereliler 2012). When pesticide concentrations decrease below the threshold value, metabolic processes are too slow to provide sufficient amounts of energy for microbial proliferation. As a result, the increase in the counts of degrading bacteria is too low to initiate the degradation process (Roch and Alexander 1997). For this reason, biological decomposition in water-bearing horizons with low phenoxy acid levels is not efficient even under aerobic conditions. In natural aquatic environment, phenoxy acids are present in concentrations lower than those used in studies of degradation of these compounds under laboratory conditions. In addition, only some types of microorganisms are adapted to use low concentrations of substrates. Therefore, degradation of the compound at low concentration levels may show differences in relation to its degradation at high concentrations (Tros et al. 1996; Gözdereliler 2012). Literature data indicate that some indigenous bacteria belonging to oligotrophs are able to metabolize low concentrations of impurities. However, there is little information in the literature about degradation of phenoxy acids at low concentrations. Gözdereliler (2012) has isolated from groundwater sediments bacterial strains that have created an effective mechanism of degradation of MCPA, present in low concentration (1 μg L^−1^). The isolated strains belonged to the genera *Proteobacteria*, *Achromobacter*, *Pseudomonas*, *Variovorax*, *Cupriavidus*, and *Sphingomonas*. The author showed that these were bacteria whose cells contained a low molecular weight nucleic acid. Bacteria of this type are well adapted to living in oligotrophic waters (Li et al. 1995).

Leachate from landfills can penetrate into groundwater (Klimek et al. 2010). The result of this process is high concentrations of phenoxy acids in the water flowing out of the landfill area (Gintautas et al. 1992). On the way of water flow from the landfill, the concentration of phenoxy acids is reduced due to dilution and various physicochemical and biological processes. In research Baun et al. (2003) at a distance of 150 m from the landfill, the concentration of MCPP decreased from 600 to 30 μg L^−1^. In studies by Tuxen et al. (2003), the presence of phenoxy acids in groundwaters under and in the vicinity of landfill was found.

The monitoring of phenoxy acids degradation by naturally occurring microorganisms expands our knowledge about self-purification processes in groundwater in the vicinity of landfills. Tuxen et al. (2003) analyzed the spontaneous degradation of phenoxy acids based on the results of chemical analyses of water and historical data relating to the geological structure and hydrogeological conditions in the area of the landfill in Sjoelund (Denmark). Phenoxy acids were identified and their concentrations in groundwater were determined to confirm degradation processes under field conditions. The chemical parameters of groundwater were determined in pure and polluted zones, and assessed whether the conditions present in the water samples tested are beneficial for degradation. Water was sampled from wells, most of which were situated along three transects: A, in the direct vicinity of the landfill (0 m); B, 50–60 m from the landfill; and C, 80–110 m from the landfill. Phenoxy acids were identified in samples from all three transects, and their concentrations were the highest in water samples from transect A. Phenoxy acid levels were clearly lower in the samples from the remaining two transects which contained oxygen. According to the authors, changes in phenoxy acid concentrations in the samples from transects B and C resulted mainly from aerobic decomposition. The redox conditions in transect A could have contributed to anaerobic degradation of phenoxy acids. Under anaerobic conditions, simple organic substances (acids, alcohols) are the first decomposition products of organic pollutants, and methane is the final decomposition product. Methane was not detected in the analyzed groundwater samples, but the presence of nonvolatile organic carbon (NVOC) was observed. According to the authors, NVOC constitutes additional evidence for the anaerobic degradation of phenoxy acids. Anaerobic decomposition probably played a more important role in the degradation of phenoxy acids in the samples from transect A than from transects B and C. This assumption was made based on higher NVOC levels in the samples from transect A as well as the presence of conditions that were more conducive to anaerobic degradation in transect A than in the remaining transects. The authors also conducted studies of ^14^C-labeled mecoprop (^14^C-MCPP) degradation using batch tests (microcosm). Sediment and water samples were collected at four different sites located in the area of the landfill. The degree of phenoxy acid mineralization was determined by measuring the radioactive activity of radioisotopes ^14^C-MCPP and ^14^CO_2_. The shortest lag phase of 15 days was noted in microcosm with the highest oxygen content (8.7 mg L^−1^). In microcosms with oxygen content of 0.3 mg L^−1^ and 3.1 mg L^−1^, lag phases did not exceed 60 days. The longest lag phase of around 145 days was reported in the oxygen-free microcosm. In all tested microcosms, 50–60% of the introduced MCPP was converted to CO_2_.

Pesticide degradation efficiency can be improved by adding electron acceptors (oxygen, nitrates), electron donors, and nutrients for the optimal growth of degrading microorganisms (Scow and Hicks 2005). Tuxen et al. (2006) also confirmed that the rate and efficiency of microbial degradation in a soil and water environment are stimulated by the amount of dissolved oxygen in water. The cited study was conducted in a laboratory on water samples from the water-bearing horizon. The samples were collected from openings drilled in two point sources of phenoxy acid pollution (Bornholm and Sjoelund in Denmark). The rate of biodegradation was monitored by measuring carbon dioxide levels. Shortening of the lag phase from 150 days to 5–25 days was correlated with an increase of the carbon dioxide concentration. The emission of ^14^CO_2_ increased to 50–70% at oxygen concentration of 7.7 mg L^−1^ in comparison with the emission level measured at oxygen concentration < 0.3 mg L^−1^ (30–50% ^14^CO_2_). The added oxygen was partially utilized to oxidize NH_4_^+^, NVOC, and organic matter bound to sediments, which is why its beneficial influence on herbicide decomposition was limited. Levi et al. (2014) analyzed the influence of added oxygen on mecoprop and dichlorprop decomposition in samples of anaerobic material from the water-bearing horizon and groundwater. Higher levels of mineralization were found, in particular for mecoprop, in the presence of oxygen. Oxygen enhanced degradation at concentrations below 2 mg L^−1^. At high oxygen concentrations (4–11 mg L^−1^), mecoprop was mineralized in 14–27% and dichlorprop in 3–9%.

The rate and efficiency of biodegradation are determined by the abundance and activity of microbial communities in the aquatic environment. Microbial responses to phenoxy acids are very important in biological purification processes. An increase in microbial counts points to a positive response to pollutants, whereas a decrease in the size of microbial populations is an indicator of the compound’s toxicity. De Lipthay et al. (2007) evaluated the influence of various doses of 2,4-D and MCPP on bacterial growth in sediments from the water-bearing horizon and the effect of the addition of trophic components on the biodegradation of the analyzed compounds in a laboratory experiment. Phenoxy acids were applied at concentrations of 1, 100, and 10,000 μg kg^−1^. At concentrations of 100 and 10,000 μg kg^−1^, 2,4-D and MCPP had a positive influence on bacterial abundance relative to control. Bacterial growth was the highest in response to 2,4-D concentration of 10,000 μg kg^−1^. The addition of nutrients stimulated herbicide mineralization at higher herbicide concentrations, and the stimulatory effect was not observed at lower herbicide concentrations. The positive effect of trophic components on mecoprop mineralization was also reported by Bestawy and Albrechtsen (2007).

Susceptibility of phenoxy acids to biodegradation can be increased by using a Fenton reaction (Sanchis et al. 2013), an electrochemical process (Fontmorin et al. 2013), or a photocatalysis (Samir et al. 2015) as a pre-treatment.

## Hydrolysis

Hydrolysis is the main chemical reaction that initiates the degradation of phenoxy acid esters in aqueous systems. During the reaction, acid forms of phenoxy acids are generated from esters of MCPA, MCPB, and 2,4-D. The rate of hydrolysis is determined by herbicide structure, water pH, and temperature. Esters of alkoxylated alcohols, in particular those with an ether bond near the –COOH group, are hydrolyzed faster than esters of aliphatic alcohols. In general, the rate of hydrolytic degradation increases at higher temperature and in alkaline water (Roberts et al. 1998; Tomlin 2006; Romero et al. 2015). Analyses of hydrolytic degradation are performed under strictly controlled temperature and pH conditions, in sterile solutions and without access to light. The half-life (DT_50_) of a herbicide’s active ingredient is calculated based on phenoxy acid concentrations measured at specific time intervals. For instance, the DT_50_ value of butoxyethyl ester 2,4-D (2,4-D BEE) at a temperature of 28 °C and pH 6 is 26 days, and it is significantly reduced to just DT_50_ = 0.6 h at pH 9. The DT_50_ values of 2-ethylhexyl ester 2,4-D (2,4-D EHE) at 25 °C are 99.7 days at pH 5, 48.3 days at pH 7, and 52.2 days at pH 9 (Table 2). The 2-ethylhexyl ester MCPA (MCPA-EHE) does not hydrolyze within a pH range of 5–7, whereas its DT_50_ value at pH 9 is < 117 h. Ester forms of phenoxy acids exhibit higher toxicity than their acid and salt forms. The stability of acid forms of phenoxy acids differs in sterile and buffered aqueous solutions. The acid forms of dichlorprop, MCPA, and 2,4-D exhibit the highest stability, whereas the acid forms of MCPP and MCPB are less stable. For instance, the DT_50_ value is estimated at 2 years for 2,4-D (EC 2001; Crane et al. 2007; Champeau and Tremblay 2013), but only 1 month for MCPP and MCPB (EC 2003; EC 2005; Champeau and Tremblay 2013).

**Table 2:** Summary of DT_50_ values for esters of phenoxy acids

Ester of phenoxy acid	Medium water type	pH	Temperature [°C]	DT_50_	Reference
Methyl ester of 2,4-D	Redistilled water	6	28	44 dni (calculated)	Zepp et al. (1975)
Methyl ester of 2,4-D	Redistilled water	9	28	1.1 h (calculated)	Zepp et al. (1975)
2-Butoxyethyl ester of 2,4-D	Redistilled water	6	28	26 dni (calculated)	Zepp et al. (1975)
2-Butoxyethyl ester of 2,4-D	Redistilled water	9	28	0.6 h (calculated)	Zepp et al. (1975)
2-Ethylhexyl ester of 2,4-D	Sterile water	5	25	99.7 dni	Concha et al. (1993)
2-Ethylhexyl ester of 2,4-D	Sterile water	7	25	48.3 dni	Concha et al. (1993)
2-Ethylhexyl ester of 2,4-D	Sterile water	9	25	52.2 dni	Concha et al. (1993)
2-Ethylhexyl ester of MCPA	Sterile buffer solution	5	n.r.	No hydrolysis	US EPA (2004)
2-Ethylhexyl ester of MCPA	Sterile buffer solution	7	n.r.	No hydrolysis	US EPA (2004)
2-Ethylhexyl ester of MCPA	Sterile buffer solution	9	n.r.	< 117 h	US EPA (2004)

*n.r.* not reported

## Photodegradation

**Fig. 2:**
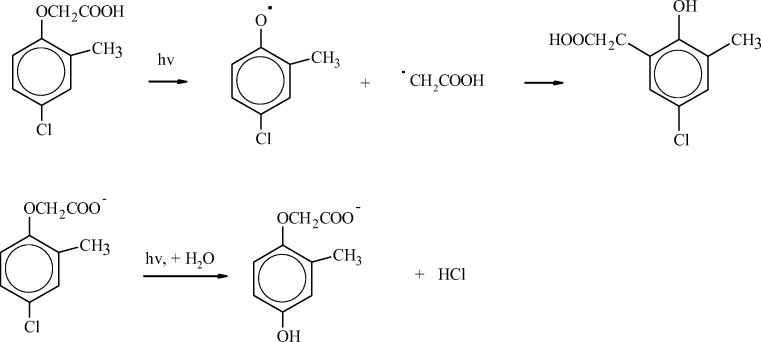
Direct photolysis processes of the protonated and deprotonated forms of MCPA (Vione 2015)

Organic compounds undergo both direct and indirect photodegradation in water (Beltman et al. 2015). Direct photodegradation takes place when molecules absorb radiation and are promoted to an excited state. The transition to an excited state initiates homolysis, heterolysis, or photoionisation reactions which lead to compound degradation (Burrows et al. 2002). The presence of aromatic structures in a phenoxy acid molecule determines a compound’s ability to selectively absorb radiation in the short wavelength range (*λ* < 290 nm). According to Vione (2015), neutral and anion forms of MCPA are characterized by a different mechanism of direct photodegradation (Fig. 2).

It should be mentioned that solar radiation reaching the Earth’s surface comprises UV radiation, primarily in the range of 290 to 400 nm (including 98% of UVA radiation with a wavelength of 315–400 nm), and UV-visible radiation (Mrzyczek 2012). Therefore, direct photodegradation of phenoxy acids exposed to solar radiation in the natural environment is barely effective (Alhousari 2011).

Indirect photodegradation can proceed via two mechanisms. The first mechanism is the oxidation of the compound by highly reactive particles (e.g., singlet oxygen ^1^O_2_ or hydroxyl radicals ^·^OH) which are generated when another radiation-absorbing substance (the photosensitizer) reacts with water and dissolved oxygen. The photosensitizer can absorb radiation with a longer wavelength than the degraded compound. The reactions involving the photosensitizer can produce a hydroxyl radical with a high potential to oxidize compounds containing an aromatic ring. In natural water bodies, hydroxyl radicals are generated in the presence of optically active dissolved organic matter (chromophoric dissolved organic matter (CDOM)) and NO_2_^−^, NO_3_^−^, and Fe^3+^ ions (Alhousari 2011). CDOM includes dissolved organic matter (DOM) compounds with particle size below 0.45 μm that interact with solar light. As suggested by Vione et al. (2006, 2010a), CDOM is more involved in the generation of ^·^OH than NO_2_^−^ and NO_3_^−^ radicals. The effectiveness of degradation can be influenced by competitive absorption of UV radiation by NO_2_^−^, NO_3_^−^, and Fe^3+^ and CDOM as well as by the compounds’ ability to scavenge hydroxyl radicals (Alhousari 2011; De Laurentiis et al. 2014).

In the surface layers of marine and inland waters, iron (III) complexes play an important role in the generation of hydroxyl radicals. In an aqueous environment, Fe^3+^ ions are hydrolyzed already at pH 3. The formation of Fe(OH)^2+^, Fe(OH)_2_^+^, Fe_2_(OH)_2_^4+^, and Fe(OH)_3_ complexes is determined by reagent concentrations and the pH of the medium. Fe(OH)^2+^ ions are the most photosensitive Fe^3+^ hydroxy complexes. Fe(OH)^2+^ is reduced and a hydroxyl radical is generated under exposure to UV radiation (Zepp et al. 1992; Brillas et al. 2009; Brillas 2014):

$$ \mathrm{Fe}{\left(\mathrm{OH}\right)}^{2+}+\mathrm{h}\upnu \to {\mathrm{Fe}}^{2+}+{}^{\cdotp}\mathrm{OH} $$

The photoreduction of Fe^3+^ to Fe^2+^ is a part of the photo-Fenton process (UV/H_2_O_2_/Fe^2+^ system). The other two reactions of the photo-Fenton process, i.e., catalytic decomposition of H_2_O_2_ induced by Fe^2+^ ions (classical Fenton reaction) and H_2_O_2_ photolysis, generate hydroxyl radicals (Klamerth et al. 2013; Clarizia et al. 2017). Fe(OH)_3_ and other insoluble iron species can be precipitated at high pH; therefore, the Fenton reaction can proceed in water with pH < 4 (Bokare and Choi 2014). In natural water bodies, H_2_O_2_ is generated through disproportionation of superoxide anion radicals (^·^O_2_^−^) which are a product of O_2_ reduction by photoexcited DOM (O’Sullivan et al. 2005). Radical generation in the Fenton process occurs mainly in inland waters that are more abundant in H_2_O_2_ and Fe^3+^ and Fe^2+^ ions than marine waters (Mopper and Zhou 1990). The main intermediates from Fenton oxidation of phenoxy acids are chlorophenols. For 2,4-D, 2,4-dichlorophenol, 2- and 4-chlorophenol, and 4-chlorocatechol were identified by Sanchis et al. (2013) and Serra-Clusellas et al. (2018) upon Fenton reaction. During the MCPA oxidation, 2- and 4-chlorophenol and 4-chlorocatechol were also formed (Sanchis et al. 2013).

Indirect photodegradation also takes place when the energy transmitted by an excited photosensitizer (e.g., CDOM in triplet state, ^3^CDOM*) promotes a compound to an excited state (Alhousari 2011). The excited compound can later undergo similar changes to those induced by direct photodegradation (Burrows et al. 2002).

Compounds that react with hydroxyl radicals relatively slowly (10^−7^ to 4 × 10^−8^ M^−1^ s^−1^) are more susceptible to direct photodegradation (Haag and Yao 1992; Alhousari 2011). The involvement of indirect photolysis in the decomposition of phenoxy acids increases in the presence of light-absorbing compounds (phenol, propan-2-ol) (Vione et al. 2010b).

Phenoxy acid esters are photodegraded at a slower rate than acid forms. The DT_50_ value of a sterile 2,4-D solution with pH 7 was determined at 13 days. In contrast, a 2,4-D 2-EHE ester solution with pH 5 was reduced by less than 15% after more than 31 days of exposure to light (APVMA 2006). Similarly to biodegradation, 4-C2MP is the main metabolite in MCPA photolysis (Kelly et al. 2019). The intermediate products of 2,4-D and mecoprop photodegradation include 2,4-dichlorophenol (Gutiérrez-Zapata et al. 2017) and 2-(4-hydroxy-2-methylphenoxy) propanoic acid (Semitsoglou-Tsiapou et al. 2016), respectively. According to Aaron et al. (2010) at lower concentration of 2,4-D, the 2-Cl and 4-Cl-phenoxyacetic acids are formed as photolytic products. However, when the 2,4-D initial concentration is higher, the additional product of photodegradation is 2,4-dichlorophenol.

The ratio of neutral to anion forms of phenoxy acid is dependent on the pH of the solution. These forms exhibit different absorption efficiency at various wavelengths (Vione et al. 2010b). The differences in radiation absorption capacity are responsible for variable rates of compound degradation in solutions with different pH. DT_50_ values were determined at 2.2 days (pH 5), 2.6 days (pH 7), and 2.4 days (pH 9) for MCPB, and at 19.5 days (river water) and 14 days (marine water) for MCPP. Under exposure to artificial radiation, DT_50_ values were estimated at 88 min (pH 5), 69 min (pH 7), and 97 min (pH 9) for buffered MCPA solutions, at 42 days (pH 5), 44 days (pH 7), and 32 days (pH 9) for MCPP, and at 4 days (pH 7) for dichlorprop (EC 2001, 2003, 2005; Crane et al. 2007; EC 2008; Champeau and Tremblay 2013). As mentioned previously, light with a wavelength of > 350 nm provides less radiation energy than light with a shorter wavelength. Furthermore, this type of radiation is absorbed by phenoxy acids to a lesser extent than UV radiation. The above explains the difference in half-life values in a photolysis reaction triggered by radiation with different wavelengths (González et al. 2018).

Photodegradation and biodegradation are processes that compete in the elimination of herbicides. However, in highly insolated and ultrapure waters, photodegradation could play a significant role in the decomposition process. Chiron et al. (2009) reported a 65% reduction in MCPA concentrations in the sunlit waters of Vaccarès lagoons. Direct photolysis of MCPA led to the formation of 4-chloro-2-methylphenol (4-C2MP). The compound then underwent photonitration to produce 4-chloro-2-methyl-6-nitrophenol (CMNP) (Chiron et al. 2009).

## Advanced photochemical methods for the elimination of phenoxy acids

The efficiency of phenoxy acid phototransformation can be enhanced through exposure to UV radiation in combination with H_2_O_2_ (Shu et al. 2013; Martinez et al. 2016; Semitsoglou-Tsiapou et al. 2016; Adak et al. 2019). Hydrogen peroxide is photolysed under exposure to UV light in an acidic environment, which leads to the generation of hydroxyl radicals (^·^OH) with high oxidation potential (Klamerth et al. 2012). Xenon-doped mercury lamps are usually used in the UV/H_2_O_2_ process. The emitted light has a wavelength of 210–240 nm with a molar absorption coefficient for H_2_O_2_ higher than at *λ* > 240 nm. The application of H_2_O_2_ in coupled processes requires an acidic compound solution. Low pH reduces H_2_O_2_ dissociation and inhibits the generation of hydroperoxyl anions (HO_2_^−^) which strongly absorb radiation and react with hydroxyl radicals (Chang and Young 2000).

Jafari and Marofi (2005) investigated 2,4-D photooxidation in a solution with pH 3.5 under exposure to H_2_O_2_ and UV lamps with different power (Table 3). In solutions containing H_2_O_2_, the mineralization of 2,4-D was completed in 120 min under exposure to a 150-W lamp and in 15 min under exposure to a 400-W lamp. In turn, in the photodegradation reaction triggered by UV radiation, 2,4-D was decomposed in 19% after 8 h of exposure to a 150-W lamp and in 99.9% under exposure to a 400-W lamp. The degradation of 2,4-D was also analyzed in acidic solutions with pH 1.5, 2.5, 3.5, and 4.5. Initial degradation proceeded at a faster rate in solutions with pH 2.5 and 3.5 than pH 4.5. Regardless of the pH of the solution, 2,4-D was degraded in 99% after 8 h of exposure to a 400-W lamp.

**Table 3:** Summary of 2,4-D removal by advanced oxidation processes

Process	Permanent conditions	Changing conditions	Time	Removal	Reference
UV/H_2_O_2_	[2,4-D] = 50 mg L^−1^, pH = 3.5	UV lamp 150 W	120 min	100%	Jafari and Marofi (2005)
		UV lamp 400 W	15 min	100%	Jafari and Marofi (2005)
UV/H_2_O_2_/micro-aeration	[2,4-D] = 100 μg L^−1^, pH = 7 [H_2_O_2_] = 10 mg L^−1^, air flow = 25 L min^−1^ UV lamp 30 W intensity 843.9 μW cm^−2^ for *λ* = 250 nm	[H_2_O_2_] = 10 mg L^−1^	60 min	> 63%	Chu et al. (2009)
		[H_2_O_2_] = 20 mg L^−1^	90 min	> 95.6%	Chu et al. (2009)
		[H_2_O_2_] = 50 mg L^−1^	60 min	> 97.2%	Chu et al. (2009)
UV/H_2_O_2_/Fe^2+^	[2,4-D] = 1 mM, pH = 2.8 [H_2_O_2_] = 1 mM, Two UV lamps intensity 1.5 10^−6^ Einstein L^−1^ s^−1^	[Fe^2+^] = 0.1 mM	60 min	77%	Kwan and Chu (2003)
UV/H_2_O_2_/Fe^3+^		[Fe^3+^] = 0.1 mM	60 min	82%	Kwan and Chu (2003)
UV/H_2_O_2_/Fe^2+^(oxalate)	[2,4-D] = 1 mM, pH = 2.8 [H_2_O_2_] = 1 mM, [oxalate] = 0.3 mM Two UV lamps intensity 1.5 10^−6^ Einstein L^−1^ s^−1^	[Fe^2+^] = 0.1 mM	60 min	77.9%	Kwan and Chu (2003)
UV/H_2_O_2_/Fe^3+^(oxalate)		[Fe^3+^] = 0.1 mM	60 min	73.6%	Kwan and Chu (2003)
UV/H_2_O_2_/Fe^3+^(oxalate)	[2,4-D] = 0.136 mM, pH = 5 [Fe^3+^] = 0.054 mM, [oxalate] = 0.54 mM UV lamp intensity 3.64 10^−8^ Einstein cm^−2^ s^−1^	H_2_O_2_/2,4-D = 7, T = 25 °C	180 min	16.4%	Schenone et al. (2015)
		H_2_O_2_/2,4-D = 50, T = 25 °C	180 min	83%	Schenone et al. (2015)
		H_2_O_2_/2,4-D = 7, T = 50 °C	180 min	63.8%	Schenone et al. (2015)
		H_2_O_2_/2,4-D = 50, T = 50 °C	180 min	95.6%	Schenone et al. (2015)
UV/TiO_2_	[2,4-D] = 45 μM, pH = 4.3 UV lamp *λ* = 254 nm	–	120 min	100%	Giri et al. (2008)
UV/TiO_2_	[2,4-D] = 40 ppm UV Lamp 4400 μW cm^−2^ for *λ* = 254 nm	–	120 min	77%	Rangel-Vasquez et al. (2015)
UV/TiO_2_-SnO_2_		0.1; 0.3; 1, 3 or 5 mol% of tin	120 min	65–93%	Rangel-Vasquez et al. (2015)
UV/TiO_2_/activated carbon system	[2,4-D] = 50 mg L^−1^, pH = 7, T = 25 °C V = 30 mL mass of TiO_2_ = 5 mg mass of carbon = 5 mg low-pressure Hg lamp 15 W Intensity 1.027 10^−4^ Einstein m^−2^ s^−1^ for *λ* = 254 nm	Untreated carbon	60 min	59–80%	Rivera-Utrilla et al. (2012)
		Carbon oxidation with O_3_ for 30 min	60 min	70%	Rivera-Utrilla et al. (2012)
		Carbon oxidation with O_3_ for 120 min	60 min	70%	Rivera-Utrilla et al. (2012)

Phenoxy acid also undergoes photodegradation in other single-phase systems where hydroxyl radicals are generated during interactions between several factors. Chu et al. (2009) demonstrated that oxygen derived from aeration in a H_2_O_2_ system can have a positive effect on the rate of 2,4-D decomposition in an acidic solution (Table 3). This effect is associated with the generation of additional amounts of ^·^OH in a reaction between ^·^O (oxygen radicals) and H^+^ ions which are present at a high concentration in an acidic solution. The source of ^·^O in the UV/H_2_O_2_/micro-aeration system was ozone homolysis at *λ* = 254 nm, and ozone was produced from oxygen at *λ* = 185 nm.

Selected organic (e.g., isopropanol) and mineral (e.g., chlorides, CO_3_^2−^, HCO_3_^−^) compounds can stimulate or inhibit the formation of hydroxyl radicals. Chu et al. (2004) observed that 2,4-D concentrations in tap water were less reduced in the UV/H_2_O_2_/micro-aeration system than in distilled and deionized water. Tap water contains Cl^−^, CO_3_^2−^, and HCO_3_^−^ ions with high rate constants in the reaction with hydroxyl radicals; therefore, they may compete for hydroxyl radicals at low concentrations of organic compounds. Anion radicals generated in the reaction exhibit considerably lower reactivity than hydroxyl radicals and are, therefore, unable to oxidize organic substances (Von Sontag et al. 1997).

Kwan and Chu (2003) demonstrated that the photo-Fenton (UV/H_2_O_2_/Fe^2+^) method and its modified versions are highly effective in degrading 2,4-D (Table 3). In the modified versions, Fe^2+^ was replaced with Fe^3+^ ions (UV/H_2_O_2_/Fe^3+^) in the reaction medium, and Fe^3+^ and Fe^2+^ oxalate complexes (UV/H_2_O_2_/Fe^2+^(oxalate) and UV/H_2_O_2_/Fe^3+^(oxalate)) were used. Iron oxalate complexes were more potent absorbers of *λ* > 200 nm radiation than Fenton’s reagent (Fe^2+^ + H_2_O_2_) (Prousek 2001; Brillas et al. 2009). The photolysis of oxalate complexes was also more efficient than the UV-induced degradation of iron hydroxy complexes. After 60 min, 2,4-D was decomposed in 77% in the UV/H_2_O_2_/Fe^2+^ system and in 82% in the UV/H_2_O_2_/Fe^3+^ system. Reduction efficiency was similar (77.9%) in the UV/H_2_O_2_/Fe^2+^(oxalate) system and somewhat lower (73.6%) in the UV/H_2_O_2_/Fe^3+^(oxalate) system. In comparison, 2,4-D reduction rates were substantially lower after 60 min in the UV (17.9%) and UV/H_2_O_2_ (33%) systems. The authors assumed that the 2,4-D decomposition rate was consistent with the kinetic model of pseudo-first-order reactions. Based on the calculated values of the degradation rate constant (*k*, pseudo-first-order reaction rate constant), the highest reduction rates were noted in the UV/H_2_O_2_/Fe^2+^(oxalate) (*k* = 2.6 × 10^−3^ s^−1^), UV/H_2_O_2_/Fe^2+^ (*k* = 1.1 × 10^−3^ s^−1^), and UV/H_2_O_2_/Fe^3+^(oxalate) (*k* = 10^−3^ s^−1^) systems. The lowest rate of decomposition was reported in the UV process (*k* = 8.5 × 10^−5^ s^−1^) (Kwan and Chu 2003). The data show that oxalate complexes of iron ions increased the rate of degradation compared with systems with uncomplexed iron. This could be related to the higher affinity of iron(III) ions to oxalate ligands than Fe(II) and, as a result, the lower ability of H_2_O_2_ activation by Fe^3+^. An important advantage of using oxalate complexes is the prevention of iron precipitation in a wider pH range and therefore the ability to conduct the Fenton process in solutions with near-neutral pH (Conte et al. 2014). According to Conte et al. (2016), the pH of the solution, which significantly determines the correct course of Fenton’s reaction, depends on the oxalate/iron ions ratio. For the 2,4-D oxidation process, an optimal oxalate/iron ions ratio of 10:1 was determined. At this ratio, the pH is below 6.5. The authors showed that under these conditions, Fe^3+^ ions are not precipitated in the form of hydroxide, while Fe(C_2_O_4_)_3_^3−^ ions constitute the dominant form of ferro-silicate complexes. Similarly, studies Schenone et al. (2015) indicated that the use of ferriooxalate complex as a source of iron enables effective degradation of 2,4-D at pH 5 (Table 3).

**Fig. 3:**
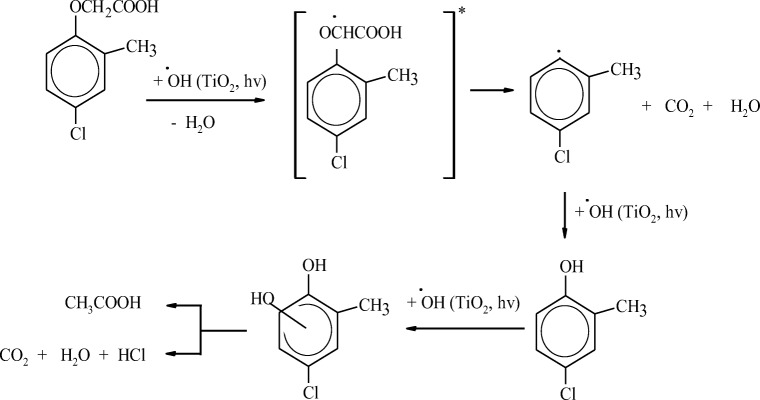
Photocatalytic degradation pathways of MCPA proposed by Topalov et al. (2001)

The photodegradation of phenoxy acids is also analyzed in two-phase systems involving UV light and a semiconductor (mostly TiO_2_) as a catalyst (Martinez et al. 2016). In the UV/TiO_2_ system, radiation with energy greater than the band gap leads to the generation of free electrons and positive sites on the surface of the photocatalyst (Ahmed et al. 2011). Hydroxyl radicals are generated when electrons are trapped by oxygen molecules, and when the positive sites of the photocatalyst react with water molecules. The efficiency of phenoxy acid degradation in the UV/TiO_2_ system can decrease with a rise in pH. This is observed past the pH value at which the surface of the TiO_2_ molecule is deprived of electric charge (pH_PZC_—point of zero charge; TiO_2_ has pH_PZC_ 6.3), which increases the ionization of phenoxy acid. At pH > pH_PZC_, the photocatalyst is negatively charged, which repels the negatively charged phenoxy acid anions (Kamble et al. 2004; Kamble et al. 2006; Wu et al. 2009; Zhou et al. 2011; Rivera-Utrilla et al. 2012). The efficiency of TiO_2_-induced photocatalysis is limited by the absorption of radiation with sufficient energy for TiO_2_ excitation. The photocatalytic activity of TiO_2_ is optimized under exposure to radiation in the wavelength range of 290 to 388 nm. Therefore, 2,4-D is more effectively degraded in the UV/TiO_2_ system at *λ* > 290 nm than just in the UV process (Guillard et al. 1994). During the photocatalytic oxidation process, 2,4-D was completely mineralized in UV/TiO_2_ and UV/ZnO systems (Djebbar and Sehili 1998) and in the solar radiation/TiO_2_ system (Herrmann et al. 1998), whereas mecoprop and MCPA were completely mineralized in the UV/TiO_2_ system (Fig. 3) (Topalov et al. 2000; Topalov et al. 2001).

The recombination of electrons with photoexcited holes on the surface of TiO_2_ molecules inhibits the generation of hydroxyl radicals. The effectiveness of the photocatalyst can be increased by adding, for example, H_2_O_2_ or O_3_ or Fe^2+^ ions. According to Farre et al. (2005), Agustina et al. (2005), and Rajeswari and Kanmani (2009), the addition of ozone to the UV/TiO_2_ system intensifies the generation of hydroxyl radicals. Giri et al. (2008) analyzed the photoactivity of TiO_2_ fiber during 2,4-D degradation (45 μM; pH ~ 4.3) under exposure to UV radiation (*λ* = 254 nm) and ozone (2 mg L^−1^ min^−1^) (Table 3). In the O_3_/UV/TiO_2_ system, 2,4-D was degraded in 100% after 2 h of exposure. In the O_3_ and UV/TiO_2_ systems, 2,4-D concentrations were reduced by 54% and 83%, respectively, after the same exposure time. In all systems, aromatic (including 2,4-dichlorophenol and benzyl alcohol) and aliphatic compounds were the intermediate decomposition products. The number of degradation products increased in the following order: UV/TiO_2_ < O_3_/UV/TiO_2_ < O_3_. An analysis of the correlation between changes in 2,4-D concentrations and the duration of exposure to UV radiation revealed that decomposition was consistent with pseudo-first-order kinetics. The highest rate of degradation was noted in the O_3_/UV/TiO_2_ system. 2,4-D was degraded at a slower rate in the UV/TiO_2_ system than in the O_3_ system. The results reported by Giri et al. (2008) are consistent with the findings of Radwan et al. (2016) who analyzed degradation of 2,4-D and MCPA in O_3_, UV/TiO_2_, and O_3_/UV/TiO_2_ systems.

During the phenoxy acid degradation processes, TiO_2_ doped with non-metals (Šojič et al. 2010; Del Ángel-Sanchez et al. 2013; Rivas et al. 2015) or modified with metal nanoparticles (Abdennouri et al. 2015; Lee et al. 2017) is also used. In this way, photocatalysts with enhanced UV activity or visible activity are obtained. Rangel-Vasquez et al. (2015) studied degradation of 2,4-D (40 ppm) in UV (intensity 4400 μW cm^−2^ at 254 nm), UV/TiO_2_, and UV/TiO_2_-SnO_2_ (TiO_2_-doped with 0.1; 0.3; 1, 3, and 5 mol% of tin) systems. After 120 min, the degree of degradation was 29% UV, 77% UV/TiO_2_, and 65–93% UV/TiO_2_-SnO_2_ (Table 3).

The methods based on the use of various photocatalysts combined with the properties of carbon adsorbents, including those chemically or physically modified, are also very effective in removing phenoxy acids. Operation with a gaseous oxidizing agent is one of the modification methods that improves the acidic and alkaline properties of the surface of activated carbons (Okoniewska 2014). Rivera-Utrilla et al. (2012) studied the degradation kinetics of 2,4-D (10, 25, 50 mg L^−1^, pH 7) in the UV/TiO_2_/activated carbon system (Table 3). A low-pressure Hg lamp with 15 W power has been used as a UV source. The studies used unmodified coals from three different producers (S, M, W) and coals that were oxidized with ozone for 30 min (WO_3-30_) and 120 min (WO_3-120_). In the UV/TiO_2_/activated carbon (WO_3-30_) system, degradation was also carried out in the presence of hydroxyl radical scavengers: t-butyl alcohol, Na_2_CO_3_, Na_2_SO_4_, and Na_2_CO_3_/Na_2_SO_4_. In comparison with UV/TiO_2_, the removal efficiency in UV/TiO_2_/activated carbon systems was significantly higher. This positive effect was connected with functioning of nanocarbon as an efficient conductor, delivering the electrons to an acceptor (Langford et al. 2014). It was shown that radical scavengers inhibited degradation in the following order: t-butyl alcohol > SO_4_^2−^ + CO_3_^2−^ > SO_4_^2−^.

Photocatalytic methods are constantly being improved in order to reduce the harm to the environment and increase their efficiency. For this purpose, research is carried out using environmentally friendly and efficient light sources. In recent years, much attention has been paid to the use of LED lamps as a source of radiation in photocatalysis. Radwan et al. (2016) compared the activity of UVA/TiO_2_ and UVA/TiO_2_/O_3_ systems in the MCPA and 2,4-D photodegradation process. The tests were carried out in a photocatalytic reactor equipped with LED diodes. It was shown that the UVA/TiO_2_/O_3_ system was characterized by a higher activity in the degradation of both phenoxy acids from the UVA/TiO_2_ system. The total distribution of MCPA and 2,4-D in the UVA/TiO_2_/O_3_ system took place in less than 40 min. Yu et al. (2013) studied the influence of the radiation source (LED and mercury lamps) on the MCPA and 2,4-D photocatalysis process carried out using TiO_2_. The tests showed greater efficiency of MCPA and 2,4-D degradation by a TiO_2_ catalyst exposed by UV LED radiation. The UV LED/TiO_2_ system also showed high efficiency in removing the mixture of herbicides. The work of Rivas et al. (2015) describes the results of MCPA degradation tests using nitrogen-doped TiO_2_ and TiO_2_ (Degussa P-25) as a reference system. A photoreactor equipped with four LED lamps (*λ* = 350–400 nm) was used in the research. The efficiency of degradation of pure MCPA in the P-25 type TiO_2_ system was higher compared with the TiO_2_ systems modified with nitrogen. Total degradation of MCPA in the TiO_2_ system (P-25) occurred after 15 min of irradiation. In the nitrogen-doped TiO_2_ systems, the total decomposition occurred in 60 to 90 min and depended on the amount of admixture in the catalyst. The authors observed a favorable effect of the increase in calcination temperature in the range of 200–600 °C on the photocatalytic activity of TiO_2_/N in the commercial degradation process of MCPA. The degradation efficiency with TiO_2_/N was lower compared with TiO_2_ (P-25). Photocatalytic oxidation of commercial MCPA was also performed using TiO_2_ samples calcined at 300 °C and containing various amounts of nitrogen admixture. It was found that doping with more N increased the TiO_2_ photoactivity. It was also shown that in the TiO_2_ (P-25) system with the addition of O_2_, the degree of degradation of pure MCPA was greater in relation to the size of the distribution observed in the presence of the following substances: propan-2-ol, KI, tert-butyl alcohol, and oxalate. According to the authors, in the conducted experiments, MCPA underwent photodegradation mainly through reactions with hydroxyl radicals, and less important in this process were reactions with hydroperoxyl and organics radicals.

## Summary

All processes that lower phenoxy acid concentrations in the water environment decrease the toxic effects of these compounds for aquatic organisms and humans. Hydrolysis, biodegradation, and photodegradation processes most efficiently reduce phenoxy acid levels in water. The efficiency of hydrolysis is determined mainly by the temperature and pH of water. Hydrolytic decomposition is observed mainly in phenoxy acid esters, whereas their acidic forms are characterized by higher hydrolytic stability. Biodegradation plays a key role in phenoxy acid decomposition in water. The rate of biological degradation is determined mainly by the qualitative and quantitative composition of microorganisms, as well as by oxygen levels in water and the properties and concentrations of phenoxy acids. In groundwater, phenoxy acid degradation involves anaerobic organisms, and the process is slower than in surface water where aerobic organisms are involved. Little is known about microorganisms that decompose phenoxy acids in water, and very few genes that encode phenoxy acid–degrading enzymes have been identified to date. Phenol derivatives are the most commonly detected biodegradation products of 2,4-D, dichlorprop, MCPA, and mecoprop. In connection with the above, it is suggested that further research is needed in order to select the microflora suitable for conducting aerobic and anaerobic biodegradation of phenoxy acids and to identify possible pathways of their degradation. Particularly little information is available regarding the degradation of phenoxy acids at low concentrations in the environment; hence, there is a need for further research in this area.

Phenoxy acids are also decomposed under exposure to sunlight. This process can play a key role in the degradation of phenoxy acids in shallow and strongly insolated waters. The results of laboratory analyses indicate that photodegradation efficiency is dependent on the form of phenoxy acids and the pH of the aqueous solution. The anionic form is more susceptible to photodegradation than phenoxy acid esters and acidic forms. The efficiency of photodegradation is also determined by the process parameters, i.e., temperature, source of radiation, radiation intensity, and wavelength. Phenoxy acids are degraded more effectively under the combined effects of UV radiation and one or combination factors (H_2_O_2_, O_2_, O_3_, TiO_2_, Fenton’s reagent Fe^2+^ + H_2_O_2_) than that under exposure to UV alone. In the scientific literature, there is little information on photodegradation of phenoxy acids in natural waters under the influence of sunlight, which would also take into account the physicochemical properties of water and the importance of soluble organic matter, especially humic acids in this process.

## References

[CR1] Aaron J-J, Guigand SI, Pejov L, Efremova-Aaron S, Zdravkovski Z (2010). Theoretical and experimental approach for the study of 2,4-dichlorophenoxyacetic acid photodegradation: C–O versus C–Cl bond dissociation energies in the gas phase and aqueous medium. Croat Chem Acta.

[CR2] Abdennouri M, Elhalil A, Farnane M, Tounsadi H, Mahjoubi FZ, Elmoubarki R (2015). Photocatalytic degradation of 2,4-D and 2,4-DP herbicides on Pt/TiO_2_ nanoparticles. J Saudi Chem Soc.

[CR3] Adak A, Das I, Mondal B, Koner S, Datta P, Blaney L (2019). Degradation of 2,4-dichlorophenoxyacetic acid by UV 253.7 and UV-H_2_O_2_: reaction kinetics and effects of interfering substances. Emerg Contam.

[CR4] Agustina TE, Ang HM, Vareek VK (2005). A review of synergistic effect of photocatalysis and ozonation on wastewater treatment. J Photochem Photobiol C: Photochem Rev.

[CR5] Ahmed S, Rasul MG, Brown R, Hashib MA (2011). Influence of parameters on the heterogeneous photocatalytic degradation of pesticides and phenolic contaminants in wastewater: a short review. J Environ Manag.

[CR6] Albrechtsen HJ, Smith PM, Nielsen P, Christensen TH (1996). Significance of biomass support particles in laboratory studies on microbial degradation of organic chemicals in aquifers. Water Res.

[CR7] Albrechtsen H-J, Clausen L, Pedersen PG (2003). Degradation of the herbicides atrazine, isoproturon and MCPP in the subsurface at four European sites. Non-agricultural use of pesticides – environmental issues and alternatives.

[CR8] Albrechtsen H-J, Mills M, Aamand J, Bjerg PL (2001). Degradation of herbicides in shallow Danish aquifers: an integrated laboratory and field study. Pest Manag Sci.

[CR9] Albrechtsen H-J, Winding A (1992). Microbial biomass and activity in subsurface sediments from Vejen, Denmark. Microb Ecol.

[CR10] Alhousari F (2011) Fate and behaviour of acidic rice herbicides in lagoon waters of Camargue (Rhône river delta, France). Ph. D. Thesis. Université de provence Aix-Marseille I http://www.theses.fr/2011AIX10022.pdf. Accessed 5 March 2019

[CR11] APVMA (2006) Australian Pesticides & Veterinary Medicines Authority. Preliminary review findings (Environment) part 1: 2,4-D esters volume 2: Technical Report. https://apvma.gov.au/sites/default/files/publication/14261-2-4-d-phase-7-prf-esters.pdf. Accessed 5 March 2019

[CR12] Arildskov NP, Pedersen PG, Albrechtsen H-J (2001). Fate of the herbicides 2,4,5-T, atrazine, and DNOC in a shallow, anaerobic aquifer investigated by in situ passive diffusive emitters and laboratory batch experiments. Groundwater.

[CR13] Batıoglu-Pazarbas M, Bælum J, Johnsen AR, Sørensen SR, Albrechtsen H-J, Aamand J (2012). Centimeter-scale vertical variability of phenoxy acid herbicide mineralization potential in aquifer sediment relates to the abundance of tfdA genes. FEMS Microbiol Ecol.

[CR14] Batıoglu-Pazarbas M, Milosevic N, Malaguerra F, Binning PJ, Albrechtsen H-J, Bjerg PL (2013). Discharge of landfill leachate to streambed sediments impacts the mineralization potential of phenoxy acid herbicides depending on the initial abundance of tfdA gene classes. Environ Pollut.

[CR15] Baun A, Reitzel LA, Ledin A, Bjerg PL, Christensen TH (2003). Natural attenuation of xenobiotic organic compounds in a landfill leachate plume (Vejen, Denmark). J Contam Hydrol.

[CR16] Beltman WHJ, Mulder HM, ter Horst MMS, Wipfler EL (2015) Transformation by photolysis in water in the pesticide model TOXSWA; Implementation report. Wageningen, Alterra Wageningen UR (University & Research centre). http://edepot.wur.nl/347914. Accessed 5 March 2019

[CR17] Bestawy EE, Albrechtsen HJ (2007). Effect of nutrient amendments and sterilization on mineralization and/or biodegradation of 14C-labeled MCPP by soil bacteria under aerobic conditions. Int Biodeterior Biodegradation.

[CR18] Boivin A, Amellal S, Schiavon M, van Genuchten MT (2005). 2,4-Dichlorophenoxyacetic acid (2,4-D) sorption an degradation dynamics in three agricultural soils. Environ Pollut.

[CR19] Bokare AD, Choi W (2014). Review of iron-free Fenton-like systems for activating H_2_O_2_ in advanced oxidation processes. J Hazard Mater.

[CR20] Boyle AW, Knight WK, Haeggblom MM, Young LY (1999). Transformation of 2,4-dichlorophenoxyacetic acid in four different marine and estuarine sediments: effects of sulfate, hydrogen and acetate on dehalogenation and side-chain cleavage. FEMS Microbiol Ecol.

[CR21] Brillas E (2014). A review on the degradation of organic pollutants in waters by UV photoelectro-Fenton and solar photoelectro-Fenton. J Braz Chem Soc.

[CR22] Brillas E, Sirés I, Oturan MA (2009). Electro-Fenton process and related electrochemical technologies based on Fenton’s reaction chemistry. Chem Rev.

[CR23] Buczyńska A, Szadkowska-Stańczyk J (2005). Identification of health hazards to rural population living near pesticide dump sites in Poland. Int J Occup Environ Health.

[CR24] Burrows HD, Canle LM, Santaballa JA, Steenken S (2002). Reaction pathways and mechanisms of photodegradation of pesticides. Invited Review J Photochem Photobiol B Biol.

[CR25] Buser H-R, Müller MD (1997). Conversion reactions of various phenoxyalkanoic acid herbicides in soil. 2. Elucidation of the enantiomerization process of chiral phenoxy acids from incubation in a D_2_O/soil system. Environ Sci Technol.

[CR26] Buss SR, Thrasher J, Morgan P, Smith JWN (2006). A review of mecoprop attenuation in the subsurface. Q J Eng Geol Hydrogeol.

[CR27] Casas ME, Nielsen TK, Kot W, Hansen LH, Johansen A, Bester K (2017). Degradation of mecoprop in polluted landfill leachate and waste water in a moving bed biofilm reactor. Water Res.

[CR28] Cassanego M, Droste A, Windisch P (2010). Effects of 2,4-D on the germination of megaspores and initial development of Regnellidium diphyllum Lindman (Monilophyta, Marsileaceae). Braz J Biol.

[CR29] Caux P-Y, Kent RA, Bergeron V, Fan GT, Macdonald DD (1995). Environmental fate and effects of MCPA: a Canadian perspective. Crit Rev Environ Sci Technol.

[CR30] Champeau O, Tremblay L (2013) Ecotoxicity review of 26 pesticides. Reporter 2357 http://www.cawthron.org.nz/media_new/publications/pdf/2013_09/CawRpt_2357_OlivierChampeau.pdf. Accessed 5 March 2019

[CR31] Chang PBL, Young TM (2000). Kinetics of methyl tert-butyl ether degradation and by-product formation during UV/hydrogen peroxide water treatment. Water Res.

[CR32] Chiron S, Comoretto L, Rinaldi E, Maurino V, Minero C, Vione D (2009). Pesticide by-products in the Rhône delta (Southern France). The case of 4-chloro-2-methylphenol and of its nitroderivative. Chemosphere.

[CR33] Chu WH, Gao NY, Li C, Cui L (2009). Photochemical degradation of typical halogenated herbicide 2,4-D in drinking water with UV/H_2_O_2_/microaeration. Sci China Ser B Chem.

[CR34] Chu W, Kwan CY, Chan KH, Chong C (2004). An unconventional approach to studying the reaction kinetics of the Fenton’s oxidation of 2,4-dichlorophenoxyacetic acid. Chemosphere.

[CR35] Clarizia L, Russo D, Di Somma I, Marotta R, Andreozzi R (2017). Homogeneous photo-Fenton processes at near neutral pH: a review. Appl Catal B Environ.

[CR36] Comoretto L, Arfib B, Talva R, Chauvelon P, Pichaud M, Chiron S (2008). Runoff of pesticides from rice fields in the Ile de Camargue (Rhône river delta, France): field study and modeling. Environ Pollut.

[CR37] Concha M, Shepler K, Erhardt-Zabik S (1993) Hydrolysis of [^14^C] 2,4-D ethylhexyl ester at pH 5, 7, and 9. PTRL Project number 387W. Unpublished study conducted by PTRL West, Inc. for Industry Task Force II on 2,4-D Research Data

[CR38] Conte LO, Querini P, Albizzati ED, Alfano OM (2014). Photonic and quantum efficiencies for the homogeneous photo-Fenton degradation of herbicide 2,4-D using different iron complexes. J Chem Technol Biotechnol.

[CR39] Conte LO, Schenone AV, Alfano OM (2016). Photo-Fenton degradation of the herbicide 2,4-D in aqueous medium at pH conditions close to neutrality. J Environ Manag.

[CR40] Crane M, Maycock D, Watts CD, Atkinson C, Johnson I (2007). Proposed EQS for water framework directive annex VIII substances: 2,4-dichlorophenoxyacetic acid (2,4-D), Science report – HOEP670085/SR15.

[CR41] DaSilva A (2016) Surface water monitoring for pesticides in agricultural areas of Northern California. https://www.cdpr.ca.gov/docs/emon/pubs/ehapreps/report_306_dasilva.pdf. Accessed 15 July 2019

[CR42] Del Ángel-Sanchez K, Vázquez-Cuchillo O, Aguilar-Elguezabal A, Cruz-López A, Herrera-Gómez A (2013). Photocatalytic degradation of 2,4-dichlorophenoxyacetic acid under visible light: effect of synthesis route. Mater Chem Phys.

[CR43] De Laurentiis E, Minella M, Maurino V, Minero C, Vione D (2014). Effects of climate change on surface-water photochemistry: a review. Environ Sci Pollut Res.

[CR44] de Lipthay JR, Aamand J, Barkay T (2002). Expression of tfdA genes in aquatic microbial communities during acclimation to 2,4-dichlorophenoxyacetic acid. FEMS Microbiol Ecol.

[CR45] de Lipthay JR, Johnsen K, Aamand J, Tuxen N, Albrechtsen H-J, Bjerg PL, Bjerg PL, Engesgaard P, Krom TD (2000). Continuous exposure of pesticides in an aquifer changes microbial biomass, diversity and degradation. Groundwater 2000, Proceedings of the International Conference on Groundwater Research, Copenhagen, 6-8 June.

[CR46] de Lipthay JR, Sørensen SR, Aamand J (2007). Effect of herbicide concentration and organic and inorganic nutrient amendment on the mineralization of mecoprop, 2,4-D and 2,4,5-T in soil and aquifer samples. Environ Pollut.

[CR47] de Lipthay JR, Tuxen N, Johnsen K, Hansen LH, Albrechtsen H-J, Bjerg PL (2003). In situ exposure to low herbicide concentrations affects microbial population composition and catabolic gene frequency in an aerobic shallow aquifer. Appl Environ Microbiol.

[CR48] Djebbar K, Sehili T (1998). Kinetics of heterogeneous photocatalytic decomposition of 2,4 dichlorophenoxyacetic acid over titanium dioxide and zinc oxide in aqueous solution. Pestic Sci.

[CR49] Dobbins DC, Aelion CM, Pfaender FK (1992). Subsurface, terrestrial microbial ecology and biodegradation of organic chemicals: a review. Crit Rev Environ Control.

[CR50] EA (2003). Pesticides 2002. The annual report of the environment agency pesticide monitoring programme.

[CR51] EC (2001) European Commission Health & Consumer Protection Directorate-General Directorate E. Food Safety: plant health, animal health and welfare, international questions E1 - Plant Heath 2,4-D 7599/VI/97-final 1 October 2001. https://www.24d.org/PDF/Regulatory_Decisions/EU/2015%20European%20Commission%20Report.pdf. Accessed 5 March 2019

[CR52] EC (2003) European Commission Health & Consumer Protection Directorate-General Directorate E – Food Safety: plant health, animal health and welfare, international questions E1 - plant heath Mecoprop SANCO/3063/99-Final 14 April 2003. http://ec.europa.eu/food/plant/pesticides/eu-pesticides-database/public/?event=activesubstance.ViewReview&id=76. Accessed 5 March 2019

[CR53] EC (2005) European Commission Health & Consumer Protection Directorate-General Directorate D. Food safety: production and distribution chain Unit D3 - chemicals, contaminants and pesticides MCPB SANCO/4063/2001-final 15 April 2005. http://ec.europa.eu/food/plant/pesticides/eu-pesticides-database/public/?event=activesubstance.ViewReview&id=197. Accessed 5 March 2019

[CR54] EC (2008) European Commission Health & Consumer Protection Directorate-General Directorate D. Food safety: production and distribution chain unit D3 - chemicals, contaminants and pesticides MCPA SANCO/4062/2001-final (11 July 2008) http://ec.europa.eu/food/plant/pesticides/eu-pesticides-database/public/?event=activesubstance.ViewReview&id=196. Accessed 5 March 2019

[CR55] EC (2006) European Union. Directive 2006/118/EC of the European Parliament and of the Council of 12 December 2006 on the protection of groundwater against pollution and deterioration. https://eur-lex.europa.eu/LexUriServ/LexUriServ.do?uri=OJ:L:2006:372:0019:0031:EN:PDF. Accessed 5 March 2019

[CR56] Ensminger MP, Budd R, Kelley KC, Goh KS (2013). Pesticide occurrence and aquatic benchmark exceedances in urban surface waters and sediments in three urban areas of California, USA, 2008–2011. Environ Monit Assess.

[CR57] EHS (2005) Environment and heritage service. Groundwater monitoring review 2004. Regional groundwater monitoring network, Northern Ireland. http://citeseerx.ist.psu.edu/viewdoc/download?doi=10.1.1.601.6932&rep=rep1&type=pdf. Accessed 5 March 2019

[CR58] Union E (2008) Directive 2008/105/EC of the European Parliament and of the Council of 16 December 2008 on environmental quality standards in the field of water policy, amending and subsequently repealing Council Directives 82/176/EEC, 83/513/EEC, 84/156/EEC, 84/156/EEC, 84/491/EEC, 86/280/EEC and amending Directive 2000/60/EC of the European Parliament and of the Council. Official Journal of the European Communities L:348/84–348/97

[CR59] Farre MJ, Franch MI, Malato S, Ayllon JA, Peral J (2005). Degradation of some biorecalcitrant pesticides by homogeneous and heterogeneous photocatalytic ozonation. Chemosphere.

[CR60] Fontmorin J-M, Fourcade F, Geneste F, Floner D, Huguet S, Amrane A (2013). Combined process for 2,4-dichlorophenoxyacetic acid treatment – coupling of an electrochemical system with a biological treatment. Biochem Eng J.

[CR61] Gamhewage M, Farenhorst A, Sheedy C (2019) Phenoxy herbicides’ interactions with river bottom sediments. J Soils Sediments. 10.1007/s11368-019-02339-x Accessed 3 September 2019

[CR62] Ghassemi M, Fargo L, Painter P, Quinlivan S, Scofield R, Takata A (1981) Environmental fates and impacts of major forest use pesticides. P. A-101-148. U.S. EPA. Office of Pesticides and Toxic Substances, Washington

[CR63] Gintautas PA, Daniel SR, Macalady DL (1992). Phenoxyalkanoic acid herbicides in municipal landfill leachates. Environ Sci Technol.

[CR64] Giri RR, Ozaki H, Taniguchi S, Takanami R (2008). Photocatalytic ozonation of 2,4-dichlorophenoxyacetic acid in water with a new TiO_2_ fiber. Int J Environ Sci Technol.

[CR65] Glozier NE, Struger J, Cessna AJ, Gledhill M, Rondeau M, Ernst WR (2012). Occurrence of glyphosate and acidic herbicides in select urban rivers and streams in Canada, 2007. Environ Sci Pollut Res.

[CR66] González GC, Julcoura C, Chaumata H, Jáuregui-Hazab U, Delmasa H (2018). Degradation of 2,4-dichlorophenoxyacetic acid by photolysis and photo-Fenton oxidation. J Environ Chem Eng.

[CR67] Gözdereliler E (2012) Groundwater bacteria: diversity, activity and physiology of pesticide degradation at low concentrations. PhD Thesis. DTU Environment Department of Environmental Engineering, Technical University of Denmark. https://orbit.dtu.dk/fedora/objects/orbit:113168/datastreams/file_9826908/content. Accessed 15 July 2019

[CR68] Guillard C, Amalric L, D’Oilveira JC, Delprat H, Hoang-Van C, Pichat P, Helz GR, Zepp RG, Crosby DG (1994). Heterogenous photocatalysis: use in water treatment and involvement in atmospheric chemistry. Aquatic and surface photochemistry.

[CR69] Gutiérrez-Zapata HM, Rojas KL, Sanabria J, Rengifo-Herrera JA (2017). 2,4-D abatement from groundwater samples by photo-Fenton processes at circumneutral pH using naturally iron present. Effect of inorganic ions. Environ Sci Pollut Res.

[CR70] Haag RW, Yao CCDJ (1992). Rate constants for reaction of hydroxyl radicals with several drinking water contaminants. Environ Sci Technol.

[CR71] Harrison I, Leader RU, Higgo JJW, Williams GM (1998). A study of the degradation of phenoxy acid herbicides at different sites in a limestone aquifer. Chemosphere.

[CR72] Heron G, Christensen TH (1992). Degradation of the herbicide mecoprop in an aerobic aquifer determined by laboratory batch studies. Chemosphere.

[CR73] Herrmann JM, Disdier J, Pichat P, Malto S, Blanco J (1998). TiO_2_ - based solar photocatalytic detoxification of water containing organic pollutants. Case studies of 2,4-dichlorophenoxyacetic acid (2,4-D) and of benzofuran. Appl Catal B Environ.

[CR74] Hiller E, Cernanský S, Zemanová L (2010). Sorption, degradation and leaching of the phenoxyacid herbicide MCPA in two agricultural soils. Pol J Environ Stud.

[CR75] Holland NT, Duramad P, Rothman N, Figgs LW, Blair A, Hubbard A (2002). Micronucleus frequency and proliferation in human lymphocytes after exposure to herbicide 2,4-dichlorophenoxyacetic acid in vitro and in vivo. Mutat Res Genet Toxicol Environ Mutagen.

[CR76] Howard PP (1991). Handbook of environmental fate and exposure data for organic chemicals, Volume III Pesticides.

[CR77] Ignatowicz K, Struk-Sokołowska J (2004). Seasonal oscillation of agrotechnical pollutants in the Narew river with especial consideration of phenoxyacetic herbicides. Annual Set The Environment Protection.

[CR78] Ignatowicz-Owsieniuk K, Skoczko I (2002). Dependence of sorption of phenoxyacetic herbicides on their physico-chemical properties. Pol J Environ Stud.

[CR79] Islam F, Farooq MA, Gill RA, Wang J, Yang C, Ali B et al (2017) 2,4-D attenuates salinity-induced toxicity by mediating anatomical changes, antioxidant capacity and cation transporters in the roots of rice cultivars. Sci Rep. 10.1038/s41598-017-09708-x 10.1038/s41598-017-09708-x PMC5585390 28874677

[CR80] Jafari AJ, Marofi S (2005). Photo-chemical degradation of 2,4-dichlorophenoxyacetic acid (2,4-D) in the effluent. J Res Health Sci.

[CR81] Janniche GS, Lindberg E, Mouvet C, Albrechtsen H-J (2010). Mineralization of isoproturon, mecoprop and acetochlor in deep unsaturated limestone and sandy aquifer. Chemosphere.

[CR82] Jørgensen LF, Stockmarr J (2009). Groundwater monitoring in Denmark: characteristics, perspectives and comparison with other countries. Hydrogeol J.

[CR83] Juhler RK, Felding G (2003). Monitoring methyl tertiary butyl ether(MTBE) and other organic micropollutants in groundwater: results from the Danish National Monitoring Program. Water Air Soil Pollut.

[CR84] Kamble SP, Deosarkar SP, Moulijn JA, Sawant SB, Pangarkar VG (2004). Photocatalytic degradation of 2,4-dichlorophenoxyacetic acid using concentrated solar radiation: batch and continuous operation. Ind Eng Chem Res.

[CR85] Kamble SP, Sawant SB, Pangarkar VG (2006). Photocatalytic mineralization of phenoxyacetic acid using concentrated solar radiation and titanium dioxide in slurry photoreactor. Chem Eng Res Des.

[CR86] Kelly J, Morrison G, Skillen N, Manesiotis P, Robertson PKJ (2019). An investigation of the role of pH in the rapid photocatalytic degradation of MCPA and its primary intermediate by low-power UV LED irradiation. Chem Eng J.

[CR87] Klamerth N, Malato S, Agüera A, Fernández-Alba AR, Maiholt G (2012). Treatment of municipal wastewater treatment plant effluents with modified photo-Fenton as a tertiary treatment for the degradation of micro pollutant sand disinfection. Environ Sci Technol.

[CR88] Klamerth N, Malato S, Agüera A, Fernández-Alba AR (2013). Photo-Fenton and modified photo-Fenton at neutral pH for the treatment of emerging contaminants in wastewater treatment plant effluents: a comparison. Water Res.

[CR89] Klimek A, Wysokiński L, Zawadzka-Kos M, Osęk M, Chrząszcz J (2010) Methodical guide in the field of PRTR for municipal waste landfills. http://www.gios.gov.pl/images/dokumenty/prtr/poradnik_20101103.pdf (in Polish). Accessed 15 July 2019

[CR90] Klingt M, Arvin E, Jensen BK (1993). Degradation of the pesticides mecoprop and atrazine in unpolluted sandy aquifers. J Environ Qual.

[CR91] Kolpin DW, Thurman EM, Linhart SM (2000). Finding minimal herbicide concentrations in groundwater? Try looking for their degradates. Sci Total Environ.

[CR92] Krzyżanowski R (2008). Application DI-SPME/GC-MS for residues analysis of (4-chloro-2-methylphenoxy) acetic acid within surface waters. Acta Sci Pol Biologia.

[CR93] Köck M, Farré M, Martínez E, Gajda-Schrantz K, Ginebreda A, Navarro A (2010). Integrated ecotoxicological and chemical approach for the assessment of pesticide pollution in the Ebro River delta (Spain). J Hydrol.

[CR94] Kurt-Karakus PB, Bidleman TF, Muir DCG, Struger J, Sverko E, Cagampan SJ (2010). Comparison of concentrations and stereoisomer ratios of mecoprop, dichlorprop and metolachlor in Ontario streams, 2006–2007 vs. 2003–2004. Environ Pollut.

[CR95] Kwan CY, Chu W (2004). A study of the reaction mechanisms of the degradation of 2,4-dichlorophenoxyacetic acid by oxalate-mediated photooxidation. Water Res.

[CR96] Kwan CY, Chu W (2003). Photodegradation of 2,4-dichlorophenoxyacetic acid in various iron-mediated oxidation systems. Water Res.

[CR97] Larsen L, Aamand A (2001). Degradation of herbicides in two sandy aquifers under redox conditions. Chemosphere.

[CR98] Lee SC, Lintang HO, Yuliati L (2017). High photocatalytic activity of Fe_2_O_3_/TiO_2_ nanocomposites prepared by photodeposition for degradation of 2,4-dichlorophenoxyacetic acid. Beilstein J Nanotechnol.

[CR99] Levi S, Hybel A-M, Bjerg PL, Albrechtsen H-J (2014). Stimulation of aerobic degradation of bentazone, mecoprop and dichlorprop by oxygen addition to aquifer sediment. Sci Total Environ.

[CR100] Li C, Grillo MP, Benet LZ (2003). In vitro studies on the chemical reactivity of 2,4 – dichlorophenoxyacetyl-S-acyl-CoA thioester. Toxicol Appl Pharmacol.

[CR101] Li WKW, Jellett JF, Dickie PM (1995). DNA distributions in planktonic bacteria stained with TOTO or TO-PRO. Limnol Oceanogr.

[CR102] Loos R, Locoro G, Comero S, Contini S, Schwesig D, Werres F (2010). Pan-European survey on the occurrence of selected polar organic persistent pollutants in ground water. Water Res.

[CR103] Loos R, Gawlik BM, Locoro G, Rimaviciute E, Contini S, Bidoqlio G (2009). EU-wide survey of polar organic persistent pollutants in European river waters. Environ Pollut.

[CR104] López-Piñeiro A, Peña D, Albarrán A, Becerra D, Sánchez-Llerena J, Fernández D (2019). Environmental fate of bensulfuron-methyl and MCPA in aerobic and anaerobic rice-cropping systems. J Environ Manag.

[CR105] Ludwig P, Gunkel W, Hühnerfuss H (1992). Chromatographic separation of the enantiomers of marine pollutants. Part 5: enantioselective degradation of phenoxycarboxylic acid herbicides by marine microorganisms. Chemosphere.

[CR106] Matamoros V, Nguyen LX, Arias CA, Salvadó V, Brix H (2012). Evaluation of aquatic plants for removing polar microcontaminants: a microcosm experiment. Chemosphere.

[CR107] Martinez S, Delgado M, Jarvis P (2016). Removal of herbicide mecoprop from surface water using advanced oxidation processes (AOPS). Int J Environ Res.

[CR108] McKnight US, Rasmussen JJ, Kronvang B, Binning PJ, Bjerg PL (2015). Sources, occurrence and predicted aquatic impact of legacy and contemporary pesticides in streams. Environ Pollut.

[CR109] McManus S-L, Richards KG, Grant J, Mannix A, Coxon CE (2014). Pesticide occurrence in groundwater and the physical characteristics in association with these detections in Ireland. Environ Monit Assess.

[CR110] Meylan WM, Howard PH (1991). Bond contribution method for estimating Henry’s law constants. Environ Toxicol Chem.

[CR111] Mithila J, Hall JC, Johnson WG, Kelley KB, Riechers DE (2011). Evolution of resistance to auxinic herbicides: historical perspectives, mechanisms of resistance, and implications for broadleaf weed management in agronomic crops. Weed Sci.

[CR112] Mopper K, Zhou X (1990). Hydroxyl radical photoproduction in the sea and its potential impact on marine processes. Science.

[CR113] Mrzyczek M (2012). Studies on synthesis and properties of 1,3-diketones.

[CR114] Müller TS, Sun Z, Kumar MPG, Itoh K, Murabayashi M (1998). The combination of photocatalysis and ozonolysis as a new approach for cleaning 2,4-dichlorophenoxyacetic acid polluted water. Chemosphere.

[CR115] Müller MD, Buser H-R (1997). Conversion reactions of various phenoxyalkanoic acid herbicides in soil. Enantiomerization and enantioselective degradation of the chiral 2-phenoxypropionic acid herbicides. Environ Sci Technol.

[CR116] NPIC (2015) National Pesticide Information Center. Pesticide fact sheet for 2,4-D. http://npic.orst.edu/factsheets/24Dgen.pdf. Accessed 5 March 2019

[CR117] Nesbitt HS, Watson JR (1980). Degradation of the herbicide 2,4-D in river water. II. The role of suspended sediment, nutrients, and water temperature. Water Res.

[CR118] Okoniewska E (2014). Changes in the properties of activated carbons on the process of modification. Proceedings of ECOpole.

[CR119] O’Sullivan DW, Neale PJ, Coffin RB, Boyd TJ, Osburn CL (2005). Photochemical production of hydrogen peroxide and methylhydroperoxide in coastal waters. Mar Chem.

[CR120] Pedersen PG (2000). Pesticide degradability in groundwater: importance of redox conditions.

[CR121] Pedersen JK, Berg PL, Christensen TH (1991). Correlation of nitrate profiles with groundwater and sediment characteristics in a shallow sandy aquifer. J Hydrol.

[CR122] Peterson MA, McMaster SA, Riechers DE, Skelton J, Stahlman PW (2016). 2,4-D Past, present, and future: a review. Weed Technol.

[CR123] PPDB (2013) The pesticide properties database (PPDB) developed by the agriculture & environment research unit (AERU), University of Hertfordshire, 2006–2013. https://sitem.herts.ac.uk/aeru/ppdb/en/atoz.htm. Accessed 5 March 2019

[CR124] Prousek J (2001). Fenton reaction for wastewater treatment chemical principles. Vlákna a textile.

[CR125] Radwan EK, Yu L, Achari G, Langford CH (2016). Photocatalytic ozonation of pesticides in a fixed bed flow through UVA-LED photoreactor. Environ Sci Pollut Res.

[CR126] Raina R, Etter ML, Buehler K, Starks K, Yowin Y (2011). Phenoxyacid herbicides in stormwater retention ponds: urban inputs. Am J Anal Chem.

[CR127] Rajeswari R, Kanmani S (2009). TiO_2_-based heterogeneous photocatalytic treatment combined with ozonation for carbendazim degradation. Iran J Environ Health Sci Eng.

[CR128] Rangel-Vázquez I, Del Angel G, Bertin V, González F, Vázquez-Zavala A, Arrieta A (2015). Synthesis and characterization of Sn doped TiO_2_ photocatalysts: effect of Sn concentration on the textural properties and on the photocatalytic degradation of 2,4-dichlorophenoxyacetic acid. J Alloys Compd.

[CR129] Reitzel LA, Tuxen N, Ledin A, Bjerg PL (2004). Can degradation products be used as documentation for natural attenuation of phenoxy acids in groundwater?. Environ Sci Technol.

[CR130] Richards K (2013) Assessment of the vulnerability of groundwater to pesticide inputs from Irish Agriculture. Project number 5784 https://www.teagasc.ie/media/website/publications/2012/5784.pdf. Accessed 5 March 2019

[CR131] Rivas J, Solis RR, Gimeno O, Sagasti J (2015). Photocatalytic elimination of aqueous 2-methyl-4-chlorophenoxyacetic acid in the presence of commercial and nitrogen-doped TiO_2_. Int J Environ Sci Technol.

[CR132] Rivera-Utrilla J, Sánchez-Polo M, Abdel daiem MM, Ocampo-Pérez R (2012). Role of activated carbon in the photocatalytic degradation of 2,4-dichlorophenoxyacetic acid by the UV/TiO_2_/activated carbon system. Appl Catal B Environ.

[CR133] Roberts TR, Hutson DH, Lee PW, Nichols PH, Plimmer JR, Roberts MC (1998) Metabolic pathways of agrochemicals. Part 1: herbicides and plant growth regulators. Royal Society of Chemistry, Cambridge

[CR134] Roch F, Alexander M (1997). Inability of bacteria to degrade low concentrations of toluene in water. Environ Toxicol Chem.

[CR135] Romero JM, Jorge NL, Grand A, Hernández-Laguna A (2015). Hydrolysis reaction of 2,4-dichlorophenoxyacetic acid. A kinetic and computational study Chem Phys Lett.

[CR136] Roseth R (2013) Plantevernmidler i grunnvann i jordbruksområder. Resultater fra prøvetaking i 2010 - 2012 (Pesticides in groundwater in agricultural areas. Results from sampling 2010-2012) Bioforsk Rapport 8: 46 (in Norwegian). https://brage.bibsys.no/xmlui/bitstream/handle/11250/2495956/Bioforsk-Rapport-2013-08-46.pdf?sequence=1&isAllowed=y. Accessed 5 March 2019

[CR137] Rügge K, Bjerg PL, Mosbæk H, Christensen TH (1999). Fate of MCPP and atrazine in an anaerobic landfill leachate plume (Grindsted, Denmark). Water Res.

[CR138] Sadowski J, Kucharski M (2006). Monitoring of cereal herbicide residues in water on arable areas. Progress in Plant Protection.

[CR139] Sadowski J, Kucharski M, Dziągwa M (2014). Influence of changes in the scope of registered plant protection products on the level of herbicide contamination of waters in agricultural areas. Progress in Plant Protection.

[CR140] Sadowski J, Kucharski M, Wujek B, Wysocki A (2009). Multiresidues of herbicides in surface and groundwater on cultivated terrain of Lower Silesia. Progress in Plant Protection.

[CR141] Samir R, Essam T, Ragab Y, Hashem A (2015) Enhanced photocatalytic–biological degradation of 2,4 dichlorophenoxyacetic acid. Bull Fac Pharm Cairo Univ 53/2: 77–82. 10.1016/j.bfopcu.2015.03.002

[CR142] Sanchis S, Polo AM, Tobajas M, Rodriguez JJ, Mohedano AF (2013). Degradation of chlorophenoxy herbicides by coupled Fenton and biological oxidation. Chemosphere.

[CR143] Sanchis S, Polo AM, Tobajas M, Rodríguez J, Mohedano AF (2014). Strategies to evaluate biodegradability: application to chlorinated herbicides. Environ Sci Pollut Res.

[CR144] Satora S, Kaczor G (2006). Changes in chemical composition of underground water from selected intakes of zapadlisko górnoslaskie. Infrastructure and Ecology of Rural Areas.

[CR145] Scheidleder A, Grath J, Winkler G, Stark U, Koreimann C, Gmeiner C (1999). Groundwater quality and quantity in Europe.

[CR146] Schenone AV, Conte LO, Botta MA, Alfano OM (2015). Modeling and optimization of photo-Fenton degradation of 2,4-D using ferrioxalate complex and response surface methodology (RSM). J Environ Manag.

[CR147] Schult J (2016) Pesticides and nutrients in groundwater of the Darwin region. Northern Territory Department of Environment and Natural Resources, Report No. 21/2016D. Palmerston. https://denr.nt.gov.au/__data/assets/pdf_file/0007/385441/Darwin_GWQ_report_final.pdf. Accessed 15 July 2019

[CR148] Scow KM, Hicks KA (2005). Natural attenuation and enhanced bioremediation of organic contaminants in groundwater. Curr Opin Biotechnol.

[CR149] Semitsoglou-Tsiapou S, Templeton MR, Graham NJD, Leal LH, Martijn BJ, Royce A (2016). Low pressure UV/H_2_O_2_ treatment for the degradation of the pesticides metaldehyde, clopyralid and mecoprop – kinetics and reaction product formation. Water Res.

[CR150] Serra-Clusellas A, De Angelis L, Lin C-H, Vo P, Bayati M, Sumner L (2018). Abatement of 2,4-D by H_2_O_2_ solar photolysis and solar photo-Fenton like process with minute Fe(III) concentrations. Water Res.

[CR151] Shu Z, Bolton JR, Belosevic M, El Din MG (2013). Photodegradation of emerging micropollutants using the medium-pressure UV/H_2_O_2_ advanced oxidation process. Water Res.

[CR152] Smith AE, Hayden BJ (1981). Relative persistence of MCPA, MCPB and mecoprop in Saskatchewan soils, and the identification of MCPA in MCPB-treated soils. Weed Res.

[CR153] Šojić D, Despotović V, Abramović B, Todorova N, Giannakopoulou T, Trapalis C (2010). Photocatalytic degradation of mecoprop and clopyralid in aqueous suspensions of nanostructured N-doped TiO_2_. Molecules.

[CR154] Song Y (2014). Insight into the mode of action of 2,4-dichlorophenoxyacetic acid (2,4-D) as an herbicide. J Integr Plant Biol.

[CR155] Stotzky G, Bollag J-M (2000). Soil biochemistry.

[CR156] Székács A, Mörtl M, Darvas B (2015). Monitoring pesticide residues in surface and ground water in Hungary: surveys in 1990–2015.

[CR157] Tayeb W, Chaieb I, Hammami M, Piotrowsky KD (2011). Environmental fate and effects of 2,4-dichlorophenoxyacetic herbicide. Herbicides: properties, crop protection and environmental hazards.

[CR158] Thorling L, Brüsch W, Hansen B, Langtofte C, Mielby S, Møller RR (2012) Grundvand. Status og udvikling 1989-2011. Teknisk rapport, GEUS 2011 (in Danish). https://www.geus.dk/media/16413/g-o-2013.pdf. Accessed 5 March 2019

[CR159] Tomlin CDS (2006). The pesticide manual: a world compendium.

[CR160] Topalov A, Abramović B, Molnár-Gábor D, Csanádi J (2001). Photocatalytic oxidation of the herbicide (4-chloro-2-methylphenoxy)acetic acid (MCPA) over TiO_2_. J Photochem Photobiol A Chem.

[CR161] Topalov A, Molnár-Gábor D, Kosanic M, Abramović B (2000). Photomineralization of the herbicide mecoprop dissolved in water sensitized by TiO_2_. Water Res.

[CR162] Toräng L, Albrechtsen H-J, Nyholm N, Bjerg PL, Engesgaard P, Krom TD (2000). Biodegradation kinetics at low concentrations (<1 μg/L) for aquifer pesticide contaminants. Groundwater 2000, Proceedings of the International Conference on Groundwater Research, Copenhagen, 6-8 June.

[CR163] Toräng L, Nyholm N, Albrechtsen H-J (2003). Shifts in biodegradation kinetics of the herbicides MCPP and 2,4-D at low concentrations in aerobic aquifer materials. Environ Sci Technol.

[CR164] Tros ME, Schraa G, Zehnder AJB (1996). Transformation of low concentrations of 3-chlorobenzoate by Pseudomonas sp. strain B13: kinetics and residual concentrations. Appl Environ Microbiol.

[CR165] Tuxen N (2002). In situ bioremediation of groundwater contaminated by herbicides from point sources.

[CR166] Tuxen T, Ejlskov P, Albrechtsen H-J, Reitzel LA, Pedersen JK (2003). Application of natural attenuation to ground water contaminated by phenoxy acid herbicides at an old landfill in Sjoelund, Denmark. Ground Water Monit Remediat.

[CR167] Tuxen N, Reitzel LA, Albrechtsen H-J, Bjerg PL (2006). Oxygen-enhanced biodegradation of phenoxy acids in ground water at contaminated sites. Ground Water.

[CR168] US EPA (2004) United States Environmental Protection Agency. Environmental fate and effects division’s risk assessment for the reregistration eligibility document for 2-methyl-4-chlorophenoxyacetic acid (MCPA). Document ID: EPA-HQ-OPP-2004-0239-0006. http://www.regulations.gov/fdmspublic/component/main. Accessed 15 July 2019

[CR169] US EPA (2005) United States Environmental Protection Agency. 2,4-dichlorophenoxyacetic acid (2,4-D) chemical summary. http://www.epa.gov/safewater/contaminants/dw_contamfs/24-d.html. Accessed 5 March 2019

[CR170] US EPA (2007) United States Environmental Protection Agency. Registration eligibility decision for Mecoprop-p (MCPP-p). https://www3.epa.gov/pesticides/chem_search/reg_actions/reregistration/red_G-53_29-Aug-07.pdf. Accessed 5 March 2019

[CR171] US EPA (2015) United States Environmental Protection Agency. National Primary Drinking Water Regulations. https://www.epa.gov/ground-water-and-drinking-water/national-primary-drinking-water-regulations#Organic. Accessed 5 March 2019

[CR172] Waite DT, Cessna AJ, Grover R, Kerr LA, Snihura AD (2002). Environmental concentrations of agricultural herbicides: 2,4-D and triallate. J Environ Qual.

[CR173] Williams GM, Harrison I, Carlick CA, Crowley O (2003). Changes in enantiomeric fraction as evidence of natural attenuation of mecoprop in a limestone aquifer. J Contam Hydrol.

[CR174] Wu RJ, Chen CC, Chen MH, Lu CS (2009). Titanium dioxide-mediated heterogeneous photocatalytic degradation of terbufos: parameter study and reaction pathways. J Hazard Mater.

[CR175] Venkov P, Topashka-Ancheva M, Georgieva M, Alexieve V, Karanov E (2000). Genotoxic effect of substituted phenoxyacetic acids. Arch Toxicol.

[CR176] Vione D, Falletti G, Maurino V, Minero C, Pelizzetti E, Malandrino M (2006). Sources and sinks of hydroxyl radicals upon irradiation of natural water samples. Environ Sci Technol.

[CR177] Vione D, Das R, Rubertelli F, Maurino V, Minero C, Pignataro B (2010). Modeling of indirect phototransformation reactions in surface waters 203–234. Ideas in chemistry and molecular sciences: advances in synthetic chemistry.

[CR178] Vione D, Khanra S, Das R, Minero C, Maurino V, Brigante M (2010). Effect of dissolved organic compounds on the photodegradation of the herbicide MCPA in aqueous solution. Water Res.

[CR179] Vione D, Minella M, Minero C (2015) Phototransformation of pesticides in the environment. https://www.researchgate.net/publication/283039485_Phototransformation_of_Pesticides_in_the_Environment. Accessed 5 March 2019

[CR180] Von Sontag C, Dowideit P, Fang X, Mertens R, Pan X, Schuchman MN (1997). The fate of peroxyl radicals in aqueous solution. Water Sci Technol.

[CR181] Yu L, Achari G, Langford CH (2013). LED-based photocatalytic treatment of pesticides and chlorophenols. J Environ Eng.

[CR182] Yuzir A, Abdullah N, Chelliapan S, Sallis P (2013). Effect of mecoprop (RS)-MCPP on the biological treatment of synthetic wastewater in an anaerobic membrane bioreactor. Bioresour Technol.

[CR183] Zepp RG, Faust BC, Hoigne J (1992). Hydroxyl radical formation in aqueous reactions (pH 3–8) of iron(II) with hydrogen peroxide: the photo-Fenton reaction. Environ Sci Technol.

[CR184] Zepp RG, Wolfe NL, Gordon JA, Bangham GL (1975). Dynamics of 2,4-D esters in surfaces water. Hydrolysis, photolysis, and vaporization. Environ Sci Technol.

[CR185] Zertal A, Molnár-Gábor D, Malouki MA, Sehili T, Boule P (2004). Photocatalytic transformation of 4-chloro-2-methylphenoxyacetic acid (MCPA) on several kinds of TiO_2_. Appl Catal B Environ.

[CR186] Zhou HY, Han J, Shams AB, Xu XH (2011). Dechlorination of 2,4-dichlorophenoxyacetic acid by sodium carboxymethyl cellulose-stabilized Pd/Fe nanoparticles. J Hazard Mater.

[CR187] Zipper C, Bolliger C, Fleischmann T, Suter MJ-F, Angst W, Müller MD et al (1999) Fate of the herbicides mecoprop, dichlorprop, and 2,4-D in aerobic and anaerobic sewage sludge as determined by laboratory batch studies and enantiomer-specific analysis. Biodegradation 10(4):271–278. 10.1023/A:1008396022622 10.1023/a:1008396022622 10633543

[CR188] Zipper C, Suter MF, Haderlein SB, Gruhl M, Kohler H-PE (1998) Changes in the enantiomeric ratio of (R)- to (S)-mecoprop indicate in situ biodegradation of this chiral herbicide in a polluted aquifer. Environ Sci Technol 32(14):2070–2076. 10.1021/es970880q

